# NPC1 as a novel therapeutic target for induction of pyroptosis in cancers

**DOI:** 10.1186/s40364-025-00823-w

**Published:** 2025-09-26

**Authors:** Chuanchao Zhang, Qiang Wang, Pan Su, Jianfei Qian, Qi Guo, Wei Wu, Rui Duan, Yufei Zhang, Youli Zu, Ling Zhong, Liuling Xiao, Qing Yi

**Affiliations:** 1 Center for Translational Research in Hematological Malignancies, Houston Methodist Cancer Center/Houston Methodist Research Institute, Houston, TX USA; 2 Department of Pathology and Genomic Medicine, Institute for Academic Medicine, Houston Methodist Research Institute, Houston, TX USA; 3 Present address: First Affiliated Hospital, School of Basic Medicine, Chongqing Medical University, Chongqing, China

**Keywords:** Cancer, NPC1, Cholesterol, Pyroptosis, U18666A

## Abstract

**Background:**

Cholesterol metabolism reprogramming is a hallmark of cancer cells that exhibit cholesterol addiction by absorbing low-density lipoprotein (LDL) to generate cholesterol for growth. Yet the underlying mechanisms remain unclear.

**Methods:**

We began by identifying Niemann-Pick C1 (NPC1) as a key cholesterol uptake gene linked to cancer progression through clinical data analysis. Using three tumor models, we showed that NPC1 promotes tumor growth by suppressing pyroptosis. Finally, we demonstrated that the NPC1 inhibitor U18666A effectively inhibits tumor growth, supporting its therapeutic potential.

**Results:**

Here we report that NPC1, a key player in cholesterol transport, protects cancer cells from pyroptosis across multiple cancer types. NPC1 expression was highly elevated in human cancers and negatively correlated with patient survival. NPC1 deficiency led to reduced cancer growth and enhanced sensitivity to pyroptosis under pyroptotic stress. NPC1 protects cancer cells from pyroptosis by maintaining cholesterol homeostasis and facilitating LDL-mediated cholesterol uptake, leading to enhanced geranylgeranyl pyrophosphate synthesis for cancer cell survival. Moreover, NPC1 inhibitor U18666A induced cancer cell pyroptosis and was highly therapeutic, either alone or combined with chemotherapeutics, against human hematologic and solid cancers in xenograft mouse models.

**Conclusion:**

This study reveals that NPC1 may be a potential therapeutic target for the treatment of human cancers.

**Supplementary Information:**

The online version contains supplementary material available at 10.1186/s40364-025-00823-w.

## Background

Metabolic reprogramming represents a hallmark of cancer cells [1]. This process involves alterations in the production, utilization, and distribution of metabolites and enables tumor cells to reconfigure their metabolic networks to support rapid proliferation and malignant phenotypes [2]. Key features of metabolic reprogramming include enhanced aerobic glycolysis (commonly known as the Warburg effect) [3], dependency on glutamine metabolism, and remodeling of lipid metabolism. Among these, cholesterol metabolic reprogramming constitutes a critical aspect of lipid metabolism alteration [4]. Accumulating evidence indicates that cancer cells exhibit a pronounced "addiction" to cholesterol [5, 6]. low-density lipoprotein (LDL), the primary carrier of cholesterol, transports cholesterol synthesized by the liver to peripheral tissues. Elevated LDL plasma concentrations, as a major cholesterol carrier, are linked to increased cancer incidence [7]. However, the underlying mechanisms remain poorly understood.

Niemann-Pick C1 (NPC1) is a well-established late endosomal membrane protein essential for the intracellular transport of cholesterol derived from LDL [8]. During cellular cholesterol uptake, the LDL receptor (LDLR) binds to LDL particles, which contain cholesterol esters, and undergoes endocytosis [9]. This process facilitates the release of free cholesterol, which is subsequently transported by NPC1 to cellular compartments such as the endoplasmic reticulum, Golgi apparatus, and plasma membrane for utilization. NPC1 deficiency disrupts intracellular cholesterol trafficking, leading to the accumulation of unesterified cholesterol and other lipids in late endosomes and lysosomes [10–12]. This dysfunction has been implicated in the pathogenesis of neurodegenerative and cardiovascular diseases [13–15]. However, the relationship between NPC1 and cancer-associated cholesterol addiction, as well as the role of NPC1 in cancer-related cholesterol metabolic reprogramming remain largely unknown.

Pyroptosis is a type of gasdermin (GSDM)-mediated cell death characterized by cell swelling and the formation of large membrane bubbles [16, 17]. GSDMs are typically maintained in an autoinhibited state through interactions between their N-terminal and C-terminal domains [18]. When pyroptosis is initiated, cleavage by specific caspases (such as caspase-1) releases the necrotic N-terminal domain, which forms oligomers and translocates to the plasma membrane to form pores [19, 20]. In recent years, numerous studies have revealed that the precise induction of pyroptosis in tumor cells can achieve potent anti-tumor effects [21–25]. Notably, cancer cells exhibit a heightened susceptibility to pyroptotic stress compared to normal cells [26]. However, how cancer cells resist this stress remains unclear. Emerging evidence suggests that metabolic reprogramming in cancer cells contributes to their resistance to pyroptosis initiation, although the underlying mechanisms have yet to be elucidated.

In this study, we identified NPC1 as a key protein for LDL uptake in cancer cells. We conducted a series of in vitro and in vivo experiments using various human cancer cell lines and murine models to investigate the role of NPC1 in cancer progression. Our findings revealed that NPC1 plays a crucial role in maintaining cholesterol homeostasis and protecting cancer cells from pyroptosis by facilitating LDL-mediated cholesterol uptake under pyroptotic stress. Both genetic and pharmacological inhibition of NPC1 effectively suppressed the development of multiple cancers. Notably, U18666A, an NPC1 inhibitor, significantly enhanced the therapeutic efficacy of conventional chemotherapy in established multiple myeloma (MM) and solid tumors, highlighting the potential for NPC1 inhibitors as promising novel chemotherapeutic agents.

## Methods

### Human patient samples

Bone marrow aspirates from patients with newly diagnosed MM or MGUS were collected at the Houston Methodist Neal Cancer Center. Bone marrow aspirates from healthy donors were purchased from Lonza Pharma&Biotech [27]. All participating patients provided written informed consent. The collection and analysis of the clinical samples were approved by the Institutional Review Board of the Houston Methodist Research Institute.

### Cell lines

Human MM cell lines ARP-1 and RPMI-8226 were cultured in RPMI-1640 medium. HepG2 (hepatocellular carcinoma), SKOV3 (ovarian cancer), MDA-MB-231 (triple-negative breast cancer), and HCT116 (colorectal cancer) cell lines were cultured in Dulbecco Modified Eagle Medium (DMEM). All cell lines were grown at 37 °C with 5% CO_2_, 1% penicillin/streptomycin and 10% fetal bovine serum. For lentivirus packaging, the human embryonic kidney cell line HEK-293 T was cultured in DMEM with 10% FBS and 1% penicillin/streptomycin at 37 °C with 5% CO_2_.

### Animal models

NSG mice (NOD.Cg-Prkdcscid Il2rgtm1Wjl/SzJ, RRID: IMSR_JAX:005557) or SCID mice (NOD.Cg-Prkdcscid/J, RRID: IMSR_JAX:001303) aged 6–8 weeks were obtained from the Jackson Laboratory. The spontaneous Vk*MYC MM model, which die of human-like MM at late age, was kindly provided by Dr. Marta Chesi (Mayo Clinic) [28] and maintained at the Houston Methodist animal facility for over one year to initiate tumorigenesis. These mice were housed and maintained in individual microisolator cages within a rack system equipped with air exchange filters. All mouse studies were approved by the Institutional Animal Care and Use Committee of the Houston Methodist Research Institute. We have complied with all relevant ethical regulations. Tumor burdens were monitored by detecting the presence of the M-band with the QuickGel SPE Kit (Helena Laboratories, Cat#: 3405) [29].

### Antibodies

For Western blot: anti-NPC1 (Abcam, Cat#: ab1341113), anti-LDLR (Proteintech, Cat#: 10,785–1-AP), anti- caspase-1 (CST, Cat#: 3866), anti-cleaved caspase-1 (CST, Cat#: 4199), anti-GSDMD (CST, Cat#: 97,558), anti-cleaved GSDMD (CST, Cat#: 36,425), anti-RhoA (Proteintech, Cat#: 10,749–1-AP), anti-actin (Proteintech, 66,009–1-Ig). Anti-mouse (NA9311ML) and anti-rabbit (NA9341ML) secondary antibodies were purchased from Cytiva. For immunofluorescence: anti-NPC1 (Abcam, Cat#: ab134113). For flow cytometry: anti-NPC1 (Abcam, Cat#: ab134113), anti-CD138-BV421 (Biolegend, Cat#: 356,516), anti-Ki67 (Thermo Fisher, Cat#: 48–5698-82).

### Reagents

U18666A (MCE, Cat#: HY-107433), Nigericin (MCE, Cat#: HY-127019), pRed-LDL (Invitrogen, Cat#: L34356), LDL (Invitrogen, Cat#: L3486), Terbinafine (MCE, Cat#: HY-17395A), Simvastatin (MCE, Cat#: HY-17502), GGPP (Sigma, Cat#: G6025), MVA (Sigma, Cat#: 43,987), Ac-YVAD-cmk (MCE, Cat#: HY-16990), Z-DEVD-FMK (MCE, Cat#: HY-12466), Necrostatin-1 (MCE, Cat#: HY-15760), Epirubicin (MCE, Cat#: HY-13624), Carfilzomib (MCE, Cat#: HY-10455), Carboplatin (MCE, Cat#: HY-17393).

### NPC1 knockdown/CRISPR knockout

For NPC1 knockdown, shRNA sequences were synthesized at Sigma (St. Louis, USA) and cloned into pLVTHM (Addgene, Cat#: 12,247). For lentivirus package, psPAX2 (Addgene, Cat#: 12,260), pMD2.G (Addgene, Cat#: 12,259), and pLVTHM-shNPC1 were mixed at 4:1:4 and transfected into HEK293T cells. After 48 h, the supernatant supplied with polybrene (5 µg/mL) was mixed 1:1 with fresh medium to infect target cells. GFP-positive cells were sorted by flow cytometry cell sorter after infection for 72 h, and knockdown efficiency was confirmed by immunoblotting. For NPC1 knockout in ARP-1, SKOV3, MDA-MB-231 and HCT116 cells, lentiCRISPR v2 (Addgene, Cat#: 52,961) was used. To obtain monoclonal NPC1 knockout cells in HepG2, cells were transfected with pX330 (Addgene, Cat#: 42,230) encoding sgRNAs and Cas9. GFP-positive cells were sorted by flow cytometry cell sorter and seeded at high dilution to generate single-cell clones. NPC1-specific shRNA: (target sequence: #1- CCACAAGTTCTATACCATATT; #2- GCCCGACTTATAGCCAGTAAT). LDLR- specific shRNA: (target sequence: GGGCGACAGATGCGAAAGAAA). NPC1-specific sgRNA: (target sequence: CGTGTTATACGGTGAAAGAG).

### Cell viability assay

Cells were seeded into 96-well plates (2000–15000 cells/well) and allowed to adhere for 12 h if they were adherent cells. For suspension cells, cells were cultured for 12 h for stabilization. The cells were then treated with specific agents for the indicated times at 37 °C in culture medium. After treatment, cells were incubated with cell viability assay reagent (Promega, Cat#: G3580) at a 10% concentration in complete medium for 2–4 h. The absorbance at 490 nm was then measured using a microplate reader. Cell survival following genotoxin exposure was determined by comparing the absorbance to that of untreated controls.

### Clonogenic assay

Clonogenic assay was performed as described previously [30]. Briefly, cells were treated with the indicated doses of U18666A for 24 h. After processing, approximately 500 adherent cells were seeded per well in a 6-well plate and subsequently incubating the cells in complete medium for 15–18 days. Colonies were then fixed and counted. For suspension cells, they were seeded at one cell per well in a 96-well plate and cultured for 15–18 days before assessing cell viability.

### Immunoblotting

Cultured cells or tumor tissues were lysed on ice for 30 min using RIPA buffer (composed of 50 mM Tris pH 8.0, 150 mM NaCl, 1% Triton X-100, 0.5% sodium deoxycholate, 1 mM EDTA, 1 mM dithiothreitol, DTT, and 1 mM PMSF). Proteins were denatured by heating them at 100 °C for 10 min in SDS loading buffer (50 mM Tris–HCl pH 6.8, 2% SDS, 10% glycerol, 0.1% bromophenol blue, and 100 mM DTT). After that, proteins were separated using SDS-PAGE and transferred onto a PVDF membrane (Millipore, Cat#: IPVH00010). The membranes were then blocked with 5% non-fat milk and incubated overnight at 4 °C with the specified primary antibodies. To detect the target proteins, HRP-conjugated secondary antibodies were applied, followed by visualization using a chemiluminescent substrate.

### Flow cytometry

Surface and PI staining was performed following the manufacturer's guidelines. For cleaved caspase 1 detection, the FAM FLICA Caspase 1 Kit (immunochemistry, Cat#: 97) or FLICA 660 Caspase 1 Kit (immunochemistry, Cat#: 9122) was used in accordance with the manufacturer's guidelines. Briefly, cells subjected to the specified treatments were resuspended in a wash buffer and incubated with the FAM-labeled FLICA probe for 30 min at 37 °C in the dark. Following staining, the cells were washed twice and analyzed using flow cytometry (BD Biosciences). For LDL-uptake assay, the experiments were performed following the instructions provided by the manufacturer. In brief, cells were maintained in lipid-free culture medium for 24 h. Subsequently, pHrodo™ Red-LDL (Thermo Fisher, Cat#: L34356) was added to the lipid-free medium at a 1:1000 dilution and incubated for the specified duration. The results were obtained using BD Fortessa X30 systems and analyzed with FlowJo_V10 software (TreeStar).

### RNA extraction and real-time quantitative RT-PCR

Total RNA was isolated using Trizol reagents (Thermo Fisher, Cat#: 15,596,026) following the manufacturer's instructions. RNA was then reverse transcribed into cDNA using the High-Capacity cDNA Reverse Transcription Kit (Invitrogen, Cat#: 4,368,814). Quantitative real-time PCR (qRT-PCR) was conducted with the QuantStudio 3 Real-Time PCR System (Invitrogen). The primers utilized in this study are: NPC1 (F: GCACCTTTTACCATCACTCCTG, R: GGCCACAGACAATAGAGCAGT), LDLR (F: TCTGCAACATGGCTAGAGACT, R: TCCAAGCATTCGTTGGTCCC), ARH (F: TTTGCATACATCGCCCAGAG, R: GGCTACGGTGAGGGTAACA), DAB2 (F: GTAGAAACAAGTGCAACCAATGG, R: GCCTTTGAACCTTGCTAAGAGA), NPC2 (F: CAAAGGACAGTCTTACAGCGT, R: GGATAGGGCAGTTAATTCCACTC).

### ELISA detection

Human kappa free light chains were detected by ELISA in accordance with the manufacturer’s instructions. Briefly, human kappa free light chain antibody (Proteintech, Cat#: A80-115A) was diluted 1:400 in PBST and incubated overnight on a 96-well Dynatech Immulon plate (Thermo Scientific, Cat#: 442,404). After blocking, mouse serum diluted 1:200 was added and incubated for one hour. The plate was washed three times with PBST, followed by incubation with Goat anti-human kappa light chain HRP Conjugate (Proteintech, Cat#: A80-115P) diluted 1:4000 in PBST for one hour. After washing, TMB substrate was used for color development, and absorbance was measured at 450 nm. The detection of mouse total IgG was performed strictly following the instructions provided in the Mouse IgG (Total) Uncoated ELISA Kit (Invitrogen, Cat#: 88–50,400-88).

### Cell swelling assay

To assess cell swelling during pyroptosis, cells were seeded in 6-well plates and treated with nigericin at a concentration of 1 µM for 24 h in combination with indicated reagents. For the LDL starvation group, ARP-1 cells were cultured in lipid-free medium (LFM) for at least 3 days. For the LDL supplementation group, cells were cultured with LFM supplemented with 20 μg/ml LDL. Following the treatment, cells were imaged using phase-contrast microscopy at 200 × magnification to capture morphological changes indicative of swelling. The percentage of cell swelling was calculated by analyzing at least 200 cells per condition.

### Cell death assays

Cell death was assessed using propidium iodide (PI) staining and trypan blue exclusion. For PI staining, cells were incubated with 5 µg/mL PI for 10 min and analyzed by flow cytometry to determine the percentage of PI-positive cells. For trypan blue exclusion, cells were mixed with trypan blue dye (1:1 ratio) and the percentage of stained (dead) cells was calculated using a Countess II Automated Cell Counter (Invitrogen).

### Intracellular cholesterol assay

Intracellular cholesterol content was measured using the Cholesterol Assay Kit (Abcam, Cat#: ab133116). Briefly, cells were homogenized in 200 µL cholesterol assay buffer and centrifuged (12,000 × g for 10 min at 4 °C) to collect supernatant. The supernatant (50 µL) was then mixed with cholesterol reaction mix (50 µL) in a 96-well plate. The mixture was incubated (60 min at 37 °C), and absorbance was measured at 570 nm.

### SPE QuickGel

Mouse tail vein blood was collected weekly, and serum was subjected to protein electrophoresis to monitor the presence of the M-band using the QuickGel SPE Kit from Helena Laboratories. Briefly, serum samples were diluted fivefold with PBS, and one μl aliquot of the diluted sample was applied to the QuickGel SPE Gel (Helena Laboratories, Cat#: 3505 T). Electrophoresis was conducted at 400 V for 5 min. Subsequently, the gel was dried using a QuickGel Chamber (Helena Laboratories) for 15 min, stained with Acid Blue for 2 min, and destained with a Destain solution.

### Quantification of mevalonate and squalene by LC–MS

sgNC and sgNPC1 ARP-1 cells were cultured in culture medium and 20–30 × 10^6^ cells were harvested for each sample for isotope tracing analysis, which was performed and analyzed by the Metabolomics Core Facility at the University of Texas MD Anderson Cancer Center. The detailed methods can be found in our previous publication [27].

### Quantification and statistical analysis

Statistical analyses were carried out using GraphPad Prism v.7.0 or Excel. Two-sided Student’s t-test was used for statistical evaluation unless otherwise stated in the figure legend. Multiple comparisons were evaluated using one-way or two-way ANOVA followed by Tukey's test. Log-rank (Mantel-Cox) test was used for survival curves between groups. Quantitative data are expressed as mean ± standard deviation (SD) or standard error of the mean (SEM). Each experiment was conducted with a minimum of three biological replicates. Statistical significance was defined as *P < 0.05, **P < 0.01, ***P < 0.001, and ****P < 0.0001.

## Results

### NPC1 expression is upregulated in human cancers and correlates with poor prognosis

**Fig. 1:**
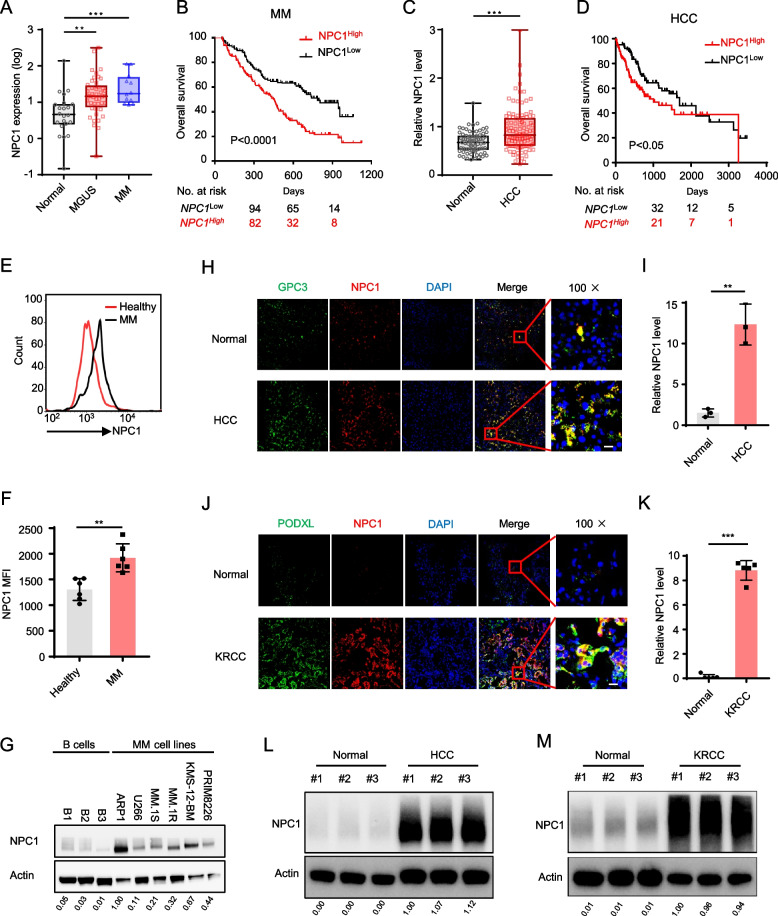
NPC1 is upregulated in various cancers and correlates with poor prognosis. Expression of *NPC1* mRNA in plasma cells of normal individual (*n* = 22) and patients with MGUS (*n* = 44) or MM (*n* = 12) (**A**) and survival analysis of MM patients with high (*n* = 132) and low (*n* = 132) NPC1 expression in Zhan MM dataset (**B**). Samples were classified by quartile expression for survival analysis. Expression of *NPC1* mRNA in normal liver (*n* = 76) and HCC tissues (*n* = 104) (**C**) and survival analysis of HCC patients with high (*n* = 101) and low (*n* = 106) NPC1 expression in Chen Liver dataset (**D**), Samples were classified by quartile expression for survival analysis. Flow cytometry showing representative NPC1 expression in plasma cells from a healthy individual and a MM patient (**E**) and summarized results (**F**) (MFI; mean fluorescence intensity) of NPC1 expression in healthy individual (*n* = 6) or MM patients (*n* = 6). Representative immunofluorescence images and summarized results of NPC1 expressions in normal liver tissues and HCC (**H**, **I**), and in normal kidney tissues and KRCC tissues (**J**, **K**), scale bar = 10 μM. Western blot analysis of NPC1 expression in normal human B cells and MM cell lines (**G**), normal liver tissues and HCC tissues (**L**), or normal kidney tissues and KRCC tissues (**M**). **P < 0.01, ***P < 0.001

We have been investigating the role of lipid metabolism in the tumor [31, 32]. Analysis of LDL uptake-related genes in human cancer cells using the Zhan et al. MM dataset [33] revealed that *NPC1*, a gene involved in cholesterol transport, was significantly overexpressed in the plasma cells of monoclonal gammopathy of undetermined significance (MGUS) and MM patients compared to those from healthy individuals (Fig. 1A). However, no significant differences were observed with other cholesterol transport related genes such as *ARH, NPC2, LDLR* or *DAB2* (Figure S1A). Moreover, MM patients with high *NPC1* expression exhibited significantly shorter overall survival than those with low *NPC1* expression (Fig. 1B). To determine whether the association of *NPC1* overexpression with poor prognosis was unique to MM, we analyzed the GEO Chen’s liver cancer dataset [34]. Consistently, *NPC1* was highly expressed in hepatocellular carcinoma (HCC) compared to normal liver tissue, and elevated *NPC1* levels were linked to poorer overall survival (Fig. 1C, D). Additionally, analysis of the TCGA dataset revealed that *NPC1* expression was upregulated across multiple cancer types (Figure S1B). Notably, high *NPC1* expression was significantly correlated with poor survival in various solid tumors (Figure S1C). Thus, these data indicate that the expression of *NPC1* is highly elevated in both hematologic and solid tumors and is associated with poor survival outcomes in patients.

To confirm the results, we examined NPC1 mRNA and protein expressions in plasma cells of healthy donors and MM patients. NPC1 protein was significantly higher in plasma cells of MM patients compared to those of healthy donors (Fig. 1E and F). Similarly, NPC1 mRNA and protein levels were markedly increased in MM cell lines compared to normal human B cells (Figs. 1G and S1D). In solid tumors, immunofluorescence staining showed that NPC1 was co-localized with tumor cells and was highly expressed in HCC and kidney renal cell carcinoma (KRCC) (Fig. 1H, I, J and K). These observations agreed with the results of Western blot and qPCR analyses (Fig. 1L, M, S1E and S1F). As NPC1 is commonly and highly expressed in human cancers, our results suggest that NPC1 may be a potential therapeutic target that warrants further investigation.

### NPC1 deficiency slows cancer growth in vitro and in vivo

**Fig. 2:**
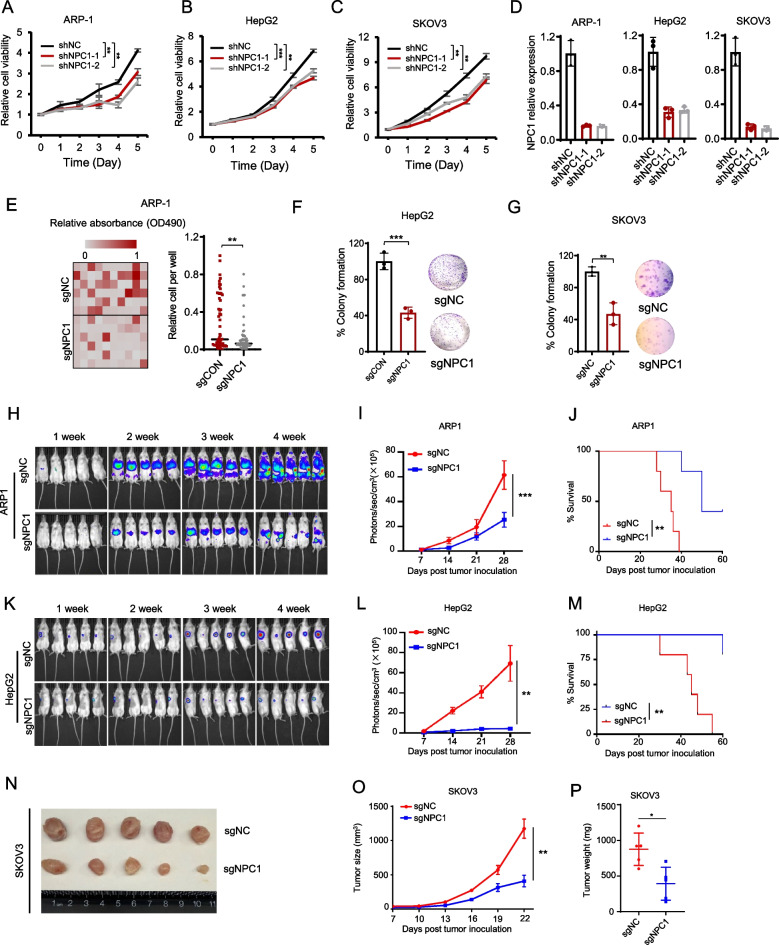
NPC1 deficiency slows cancer growth in vitro and in vivo. After transfection with the indicated shRNAs, the growth of ARP-1 (**A**), HepG2 (**B**) and SKOV3 (**C**) cells was measured by cell viability assay. NPC1 knockdown efficacy in ARP-1 cells is shown in (**D**). Results (representative images and summarized results) of colony formation in ARP-1 (**E**), HepG2 (**F**), and SKOV3 (**G**) cells after transfection with control (CON) or NPC1 sgRNA. Tumor burdens showing as bioluminescent images of female NSG mice (*n* = 5) bearing ARP-1-luc cells expressing sgNC and sgNPC1 (**H**), or HepG2-luc cells (**K**) after tumor inoculation. Summarized results of bioluminescent signals of ARP-1-luc (**I**) and HepG2-luc (**L**) cell-bearing NSG mice. Survival of ARP-1-luc (**J**) and HepG2-luc (**M**) cell-bearing NSG mice. Images of tumor masses from NSG mice bearing SKOV3 cells expressing sgNC and sgNPC1 on day 22 after tumor inoculation (**N**), summarized tumor burdens throughout the experiment process (**O**), and tumor weights from the mice on day 22 after tumor inoculation (**P**). *P < 0.05, **P < 0.01, ***P < 0.001

To explore the role of NPC1 in human cancers, we knocked down NPC1 in three human cancer cell lines. Compared to negative control (shNC), NPC1 knockdown significantly suppressed cell growth in MM (ARP-1), hepatocellular carcinoma (HepG2) and ovarian cancer (SKOV3) cells in vitro (Fig. 2A-D). Consistently, NPC1 knockdown reduced Ki67 levels in ARP-1, HepG2, and SKOV3 cells (Figures S2A-S2C). To validate the results, we used the CRISPR/sgRNA system to deplete NPC1 in different human cancer cell lines and observed a decreased cell growth in MM ARP-1 and RPMI-8226 (another MM cell line), HepG2, SKOV3, colon cancer HCT116, and breast cancer MDA-MB-231 cells (Figures S2D, S2E). Furthermore, colony-forming assay indicated a diminished colony formation ability in various cancer cells following NPC1 depletion (Figs. 2E-G and S2F-S2G). These data indicate that NPC1 deficiency suppresses tumor cell growth in vitro.

We then investigated the role of NPC1 in regulating tumor development in vivo using human xenograft mouse models. We conducted experiments in both hematologic (MM) and solid tumor models, including HCC and ovarian cancer. We injected luciferase-expressing negative control (sgNC) or NPC1-depleted (sgNPC1) ARP-1 cells into NSG mice via the tail vein and monitored tumor growth. NPC1 depletion led to a drastic reduction in tumor burden (Fig. 2H, I) and impaired tumor infiltration into the bone marrow (tumor bed) (Figures S2H). Moreover, NPC1 depletion led to a significant improvement in the survival of tumor-bearing mice (Fig. 2J). Similar phenomena were observed in solid tumors including HepG2 (Fig. 2K–M) and SKOV3 (Fig. 2N–P). Thus, these data revealed that NPC1 may play an important role in cancer development and progression.

### NPC1 protects cancer cells from pyroptosis cell death

**Fig. 3:**
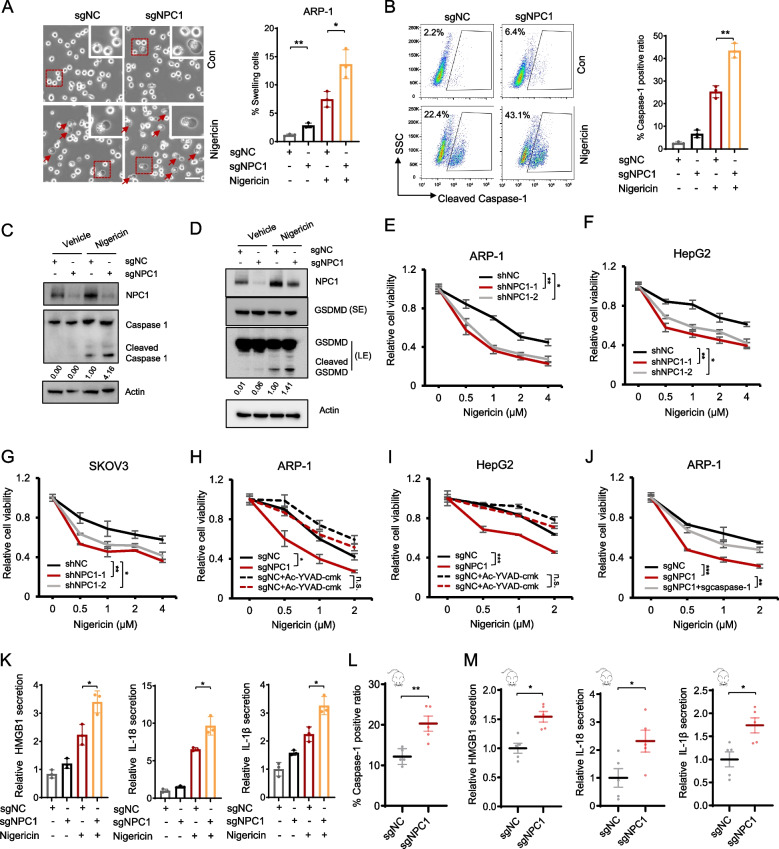
NPC1 protects cancer cells from pyroptosis cell death. Representative images of ARP-1 cells showing cell swelling in the indicated conditions (left panels) and summarized results (right panel) showing the proportion of cell swelling observed in > 200 cells, scale bar = 30 μM (**A**). Cleaved caspase-1 was examined in indicated ARP-1 cells by flow cytometry and the percentages of caspase-1-positive cells were quantified in NPC1-depleted ARP-1 cells treated with 10 μM nigericin for 4 h (**B**). Western blot showing the levels of caspase-1/cleaved caspase-1 (**C**) and GSDMD/cleaved GSDMD (**D**) in NPC1-depleted ARP-1 cells. Cell viability of ARP-1 (**E**), HepG2 (**F**), and SKOV3 (**G**) cells transfected with the indicated shRNAs and treated with the indicated doses of nigericin for 24 h. Cell viability of the indicated ARP-1 (**H**) and HepG2 (**I**) cells treated with or without Ac-YVAD-cmk (20 μM) in response to different doses of nigericin for 24 h. Cell viability of ARP-1 cells transfected with the indicated shRNAs and treated with the indicated doses of nigericin for 24 h (**J**). ELISA showing the levels of HMGB1, IL-18, and IL-1β in NPC1-depleted ARP-1 cells treated with nigericin for 24 h (**K**). Flow cytometry of cleaved caspase-1 levels in sgNC and NPC1-depleted ARP-1 cells isolated from mice (**L**). ELISA showing the levels of HMGB1, IL-18, and IL-1β in sgNC and NPC1-depleted ARP-1 cells isolated from mice (**M**). *P < 0.05, **P < 0.01, ***P < 0.001

We elucidated the mechanisms underlying NPC1-mediated growth regulation of cancer cells. Interestingly, NPC1-depleted cancer cells exhibited a slight increase in cell death (Figure S3A-S3B), and NPC1-depleted ARP-1 cells displayed a swelling phenotype, which is a characteristic feature of pyroptosis, and nigericin (as an inducer of pyroptosis) treatment increased the numbers of swelling cells (Fig. 3A). In line with the increased number of swelling cells, NPC1 depletion markedly upregulated the expression of pyroptosis marker proteins such as cleaved caspase-1 and gasdermin D (GSDMD), while the total protein levels of NLRP3, caspase-1, GSDMD, and ASC were not significantly altered (Figs. 3B-D and S3C). Moreover, nigericin induced more cell death in NPC1-deficient ARP-1 cells in a time-dependent fashion (Figure S3D). Nevertheless, nigericin did not induce the expression of the apoptosis marker cleaved caspase-3 (Figure S3E). These results suggest that NPC1 may be involved in the pyroptosis of cancer cells.

To confirm the results, NPC1-depleted ARP-1, HepG2, and SKOV3 cells were treated with the pyroptosis inducer nigericin, and cell viability was examined. NPC1 depletion significantly sensitized the cells to nigericin-induced pyroptosis (Fig. 3E-G). Likewise, when we switched to the sgRNA system to deplete NPC1, we observed an increased sensitivity to nigericin in ARP-1, HepG2, SKOV3, MDA-MB-231 and HCT116 cells (Figure S3F). Furthermore, inhibition of caspase 1 activity by the small molecule Ac-YVAD-CMK abolished the increased sensitivity of NPC-1-depleted ARP-1 and HepG2 cells to nigericin (Fig. 3H, I). Moreover, we found that caspase-1 knockout also abolished the increased sensitivity of NPC1-depleted ARP-1 cells to nigericin (Figs. 3J and S3G). Notably, pyroptosis triggers the release of danger-associated molecular patterns such as HMGB1 and involves caspase-1–mediated maturation of IL-1β and IL-18 [35]. Consistent with enhanced pyroptotic activity, NPC1 deficiency resulted in elevated release of HMGB1, IL-18, and IL-1β following nigericin stimulation (Fig. 3K). These findings indicate that NPC1 deficiency sensitizes cells to the induction of pyroptosis by promoting caspase-1 activation under pyroptotic stress such as nigericin treatment.

To confirm that pyroptosis occurred in NPC1-depleted cancer cells in vivo, we injected negative control (sgNC) or NPC1-depleted (sgNPC1) ARP-1 cells into SCID mice and monitored tumor growth. After 21 days, mice were euthanized, and cleaved caspase-1 in bone marrow ARP-1 cells was analyzed by flow cytometry (Figure S3H). As expected, the level of IgA kappa light chain indicated that tumor burden was lower in the sgNPC1 group (Figure S3I), and we observed that cleaved caspase-1 level was increased in NPC1-depleted ARP-1 cells from MM-bearing mouse bone marrow (Fig. 3L). Similarly, levels of HMGB1, IL-18, and IL-1β were elevated in the bone marrow under NPC1-deficient conditions (Fig. 3M)*.* Thus, these results strongly indicate that NPC1 protects cancer cells from pyroptosis cell death.

### Cancer cells require NPC1 for an enhanced LDL uptake to resist pyroptosis

**Fig. 4:**
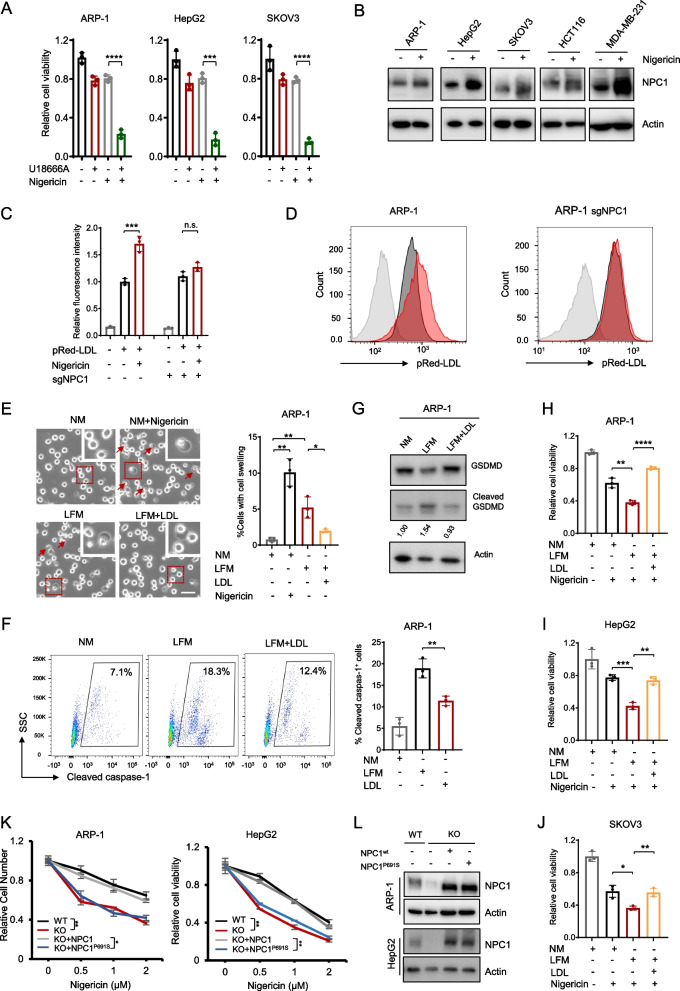
Cancer cells require NPC1 for an enhanced LDL uptake to resist pyroptosis. Cell viability of ARP-1, HepG2, and SKOV3 cells treated with U18666A (10 µM) in combination of nigericin (1 µM) for 24 h (**A**). Western blot analysis showing the expression levels of NPC1 in different cells after the treatment with 1 µM nigericin for 24 h (**B**). Fluorescence intensity (summarized results in left panel and flow staining in right) of ARP-1 (**C**) and NPC1-depleted ARP-1 (**D**) cells treated with 1 μM nigericin in the presence of fluorescence-labeled pRed-LDL for 24 h. Representative images (left panels) and summarized results (right panel) of ARP-1 cells showing cell swelling after continuous culture in normal medium (NM), lipid-free medium (LFM), and LFM + LDL (20 μg/ml) for three days. Cells treated with 1 μM nigericin for 24 h served as a positive control, scale bar = 30 μm (**E**). ARP-1 cells cultured in NM, LFM, and LFM + LDL (20 μg/ml) medium examined by flow cytometry and the percentages of caspase-1-positive cells are shown (**F**). Western blot showing the expression of GSDMD and cleaved GSDMD proteins in ARP-1 cells cultured in NM, LFM, and LFM + LDL (20 μg/ml) medium (**G**). Cell viability of ARP-1 (**H**), HepG2 (**I**), and SKOV3 (**J**) cells cultured under the indicated conditions. The values shown for each group are the ratios relative to the untreated control for each group and standardized to the NM group. Cells were treated with 1 μM nigericin for 24 h. NPC1/NPC1P691S-supplemented NPC1-KO HepG2 cells were treated with 1 μM nigericin, followed by cell viability assay (**K**). Western blot showing the expression of NPC1 in the indicated HepG2 cells (**L**). *P < 0.05, **P < 0.01, ***P < 0.001, ****P < 0.0001

To investigate whether the protective effect of NPC1 against pyroptosis depends on its function in LDL uptake, we treated cancer cells with U18666A, which is a selective inhibitor that blocks the function of NPC1 protein leading to impaired cellular uptake of LDL [36]. We observed that U18666A did not affect NPC1 protein expression (Figure S4A) but enhanced the sensitivity of ARP-1, HepG2 and SKOV3 cells to nigericin (Fig. 4A). To explore whether LDL uptake is involved in pyroptosis, we treated ARP-1 cells with nigericin and examined the expression of genes related to LDL uptake pathway. Results showed that the expression of NPC1 was significantly elevated in a dose-dependent manner following nigericin treatment (Figure S4B and S4C), and the phenomenon was observed in various cancer cell lines (Fig. 4B). Interestingly, adding fluorescent LDL to the medium enabled ARP-1 cells to actively take up more LDL under nigericin treatment, and this activity was abolished in NPC1-depleted cells (Fig. 4C, D). A similar phenomenon was observed in HepG2 cells (Figures S4D and S4E).

We conducted a cell swelling experiment to confirm the role of LDL in cancer cell pyroptosis. ARP-1 cells cultured in lipid-free medium (LFM) underwent significantly more pyroptotic cell death compared to other groups by displaying cell swelling with large bubbles in the plasma membrane, while adding LDL reversed LFM-induced pyroptosis. Cancer cells treated with nigericin in normal medium were used as a positive control (Fig. 4E). Similar results were obtained with HepG2 cells (Figure S4F). Consistently, LDL supplement rescued LFM-induced cleaved caspase-1 and GSDMD expression, as well as cell death (Figs. 4F, G and S4G). Similarly, the caspase-1 inhibitor Ac-YVAD-cmk significantly rescued LFM-induced cell death (Figure S4H). Interestingly, we found that LFM enabled ARP-1, HepG2 and SKOV3 cells to be more sensitive to nigericin, while adding LDL to the medium reversed this effect (Fig. 4H-J). These results indicate that cancer cells are prone to pyroptosis in a lipid-deficient environment and LDL supplement protects cancer cells from pyroptosis.

To confirm that NPC1 protected cancer cells from pyroptosis by LDL uptake, we generated a NPC1 knockout (NPC1-KO) ARP-1 and HepG2 cells and reintroduced the wild-type (WT) or P691S mutant (NPC1^P691S^, which reduces NPC1 cholesterol transport [37]) NPC1 using lentivirus to the NPC1-KO cells. NPC1^P691S^ failed to rescue the enhanced sensitivity of NPC1-KO cells to nigericin, while WT NPC1 did (Fig. 4K, L). Taken together, these data indicate that cancer cells require NPC1 to enhance LDL uptake to resist pyroptosis.

### NPC1 deficiency-mediated pyroptosis is dependent on the mevalonate pathway metabolite

**Fig. 5:**
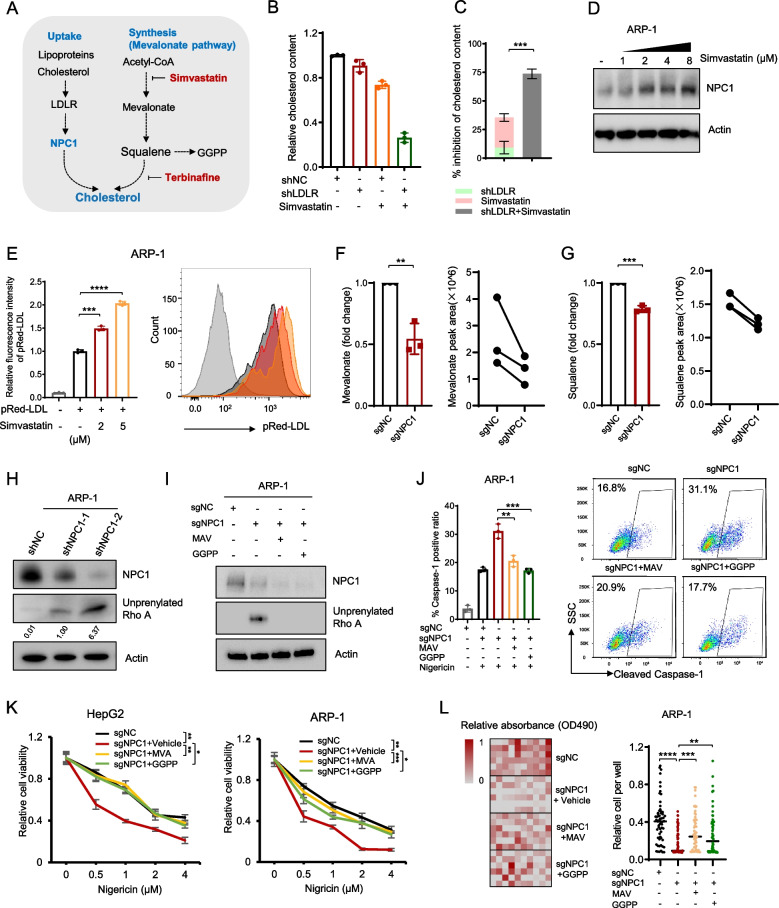
NPC1 deficiency-mediated pyroptosis is dependent on the mevalonate pathway metabolite. Cholesterol metabolism pathway diagram (**A**). Detection of cholesterol content in indicated ARP-1 cells, LDLR is a key gene in the LDL uptake pathway, and shLDLR inhibits LDL uptake. **B** Comparison of the combined inhibition rate in LDLR knockdown and simvastatin (2 μM for 24 h) treatment versus the inhibition rate by simultaneous LDLR knockdown and simvastatin treatment in ARP-1 cells (**C**). ARP-1 cells were treated with nigericin at the indicated dose for 24 h, and the expression level of NPC1 was tested by Western blot (**D**). Fluorescence intensity of pRed-LDL detected by flow cytometry in ARP-1 cells treated with the indicated dose of simvastatin for 24 h in the presence of fluorescence-labeled pRed-LDL (**E**). sgNC or sgNPC1 ARP-1 cells were counted and harvested, followed by detection of mevalonate (**F**) or squalene (**G**) levels in the cells by HPLC–MS. Unprenylated RhoA level in NPC1-depleted ARP-1 cells was measured by Western blot (**H**). Level of unprenylated RhoA in NPC1-depleted ARP-1 cells measured by Western blot with or without MVA (100 µM) or GGPP (10 µM) (**I**). Flow cytometry detection of cleaved caspase-1 levels in indicated ARP-1 cells with or without MVA (100 µM) or GGPP (10 µM) (**J**). Cell viability of ARP-1 and HepG2 cells treated with the indicated concentration of nigericin, with or without MVA (100 µM) or GGPP (10 µM) (**K**). Clonogenic activity of NPC1-depleted ARP-1 cells with or without MVA (100 µM) or GGPP (10 µM) (**L**). *P < 0.05, **P < 0.01, ***P < 0.001, ****P < 0.0001

To determine the underlying molecular mechanism of how NPC1 regulates cancer cell pyroptosis, we integrated NPC1-associated LDL/cholesterol metabolism genes with a list of pyroptosis-related genes predicted by published studies [38–41]. By examining the intersection of these two gene sets, we narrowed down the range of pyroptosis-related pathways influenced by NPC1 and identified eight relevant genes. Interestingly, six of these genes belong to the mevalonate (MVA) pathway (Figure S5A). The MVA pathway synthesizes cholesterol, with its primary end products being cholesterol or geranylgeranyl pyrophosphate (GGPP) [42–44] (Fig. 5A). GGPP is essential for RhoA prenylation [45]. Lack of GGPP inhibits RhoA prenylation and triggers inflammasome assembly, thereby leading to pyroptosis [35, 46–48]. Using simvastatin to target the MVA pathway and reduce GGPP synthesis, we found that simvastatin treatment increased nigericin sensitivity in ARP-1, HepG2, and SKOV3 cells, highlighting a role of MVA pathway in inhibiting pyroptosis (Figures S5B-S5D). Cancer cells, characterized by “cholesterol addiction” due to their high metabolic demands, obtain cholesterol either through uptake or de novo synthesis [5, 49]. Thus, we hypothesized that cancer cells adapt to pyroptotic stress by prioritizing GGPP synthesis over cholesterol and compensating for reduced de novo cholesterol synthesis through an increased uptake to maintain intracellular cholesterol levels to support their proliferation. NPC1 deficiency drives increased cholesterol synthesis via the MVA pathway, thereby reducing GGPP production and accelerating the consumption of pathway intermediates.

To test our hypothesis, we evaluated the balance between cholesterol uptake and synthesis. By individually or concurrently inhibiting these pathways, we quantified intracellular cholesterol levels. Inhibition of cholesterol uptake resulted in a modest 9.2% reduction in intracellular cholesterol, while synthesis inhibition led to a 26.3% reduction. However, concurrent inhibition of both pathways resulted in a synergistic reduction of 73.7% (Fig. 5B and C), indicating a compensatory interaction between cholesterol uptake and synthesis. Interestingly, inhibition of cholesterol synthesis using simvastatin or terbinafine upregulated NPC1 protein levels (Figs. 5D and S5E). Moreover, increased cellular LDL uptake was observed in ARP-1, HepG2, and SKOV3 cells treated with simvastatin (Figs. 5E, S5F and S5G), and this effect was abolished in NPC1-KO HepG2 cells (Figure S4H), further supporting the existence of a cholesterol balance system.

Next, we examined the effects of disrupting this balance by knocking down NPC1. As expected, NPC1 depletion in ARP-1 cells resulted in reduced MVA and squalene levels (Fig. 5F, G). This suggests that more intermediates in the MVA pathway are consumed at an increased rate for cholesterol synthesis. Since GGPP is essential for RhoA prenylation, we examined unprenylated RhoA levels. Simvastatin treatment caused a dose-dependent increase in unprenylated RhoA (Figure S5I) and unprenylated RhoA was also elevated in NPC1-depleted ARP-1 cells (Fig. 5H). Consistently, LFM treatment increased unprenylated RhoA levels, which was rescued by adding LDL to the medium (Figure S5J). Similarly, supplementation with MVA or GGPP restored unprenylated RhoA levels in NPC1-depleted cells (Fig. 5I). Notably, MVA and GGPP complementation also reversed the increases in cleaved caspase-1 levels and nigericin sensitivity in NPC1-depleted cells (Figs. 5J–L and S5K). Thus, these findings suggest that cancer cells adapt to pyroptotic stress by increasing LDL uptake and prioritizing GGPP synthesis through the MVA pathway over cholesterol production, thus enhancing their resistance to pyroptosis induction.

### NPC1 inhibitors are potential anti-tumor agents

**Fig. 6:**
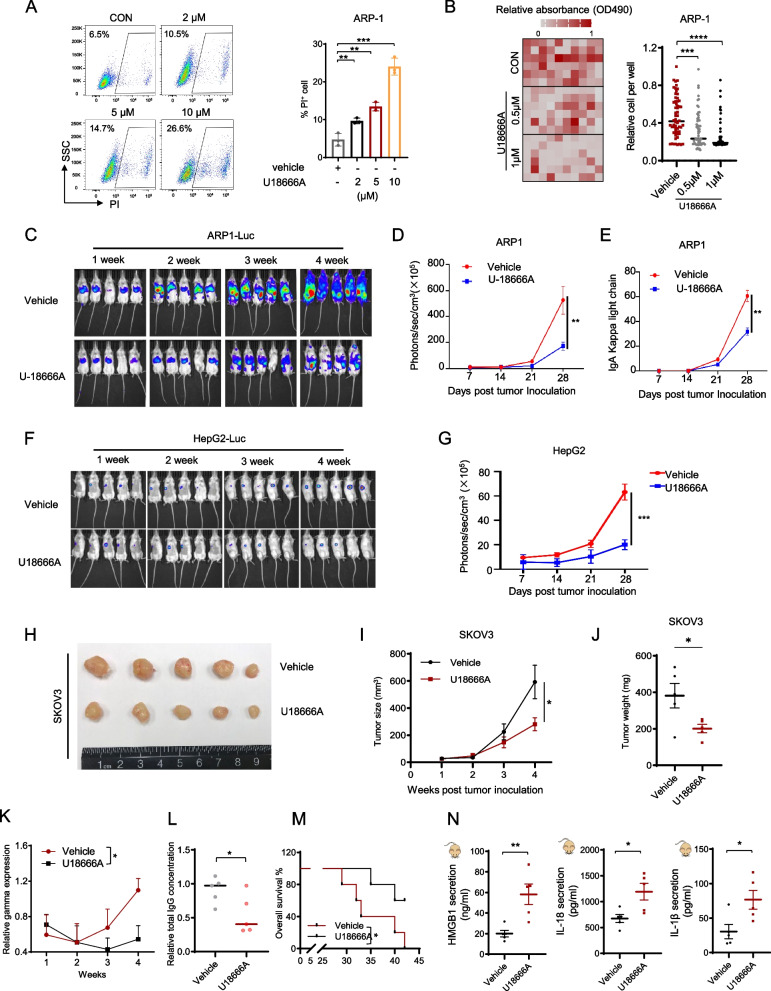
Targeting NPC1 by U18666A is therapeutic against cancer. Percentages (representative images in left panels and summarized results in right) of PI-positive ARP-1 cells cultured with the indicated U18666A concentrations for 72 h examined by flow cytometry (**A**). Clonogenic activity (representative images in left panels and summarized result in right) of NPC1 ARP-1 cells with or without the indicated dose of U18666A (**B**). Bioluminescent images of ARP-1-luc (**C**) or HepG2-luc (**F**) cell-bearing female NSG mice treated with 5 mg/kg U18666A or vehicle collected after tumor inoculation. Summarized results of bioluminescent signals of ARP-1-luc (**D**) or HepG2-luc (**G**) cell-bearing female NSG mice after tumor inoculation. ELISA was used to detect the levels of IgA kappa light chain in ARP-1 cell-bearing mice (**E**). Tumor images of NSG mice bearing SKOV3 cells with or without U18666A treatment on week 3 after tumor inoculation (**H**); tumor sizes throughout the process (**I**); and tumor weights on week 3 after tumor inoculation (**J**). Tumor burdens as detected by quantification of the SPE QuickGel Gamma region (**K**). ELISA for total IgG levels in mice treated with or without U18666A (**L**). Overall survival of mice treated with or without U18666A (**M**). ELISA for HMGB1, IL-18 and IL-1β in mice treated with or without U18666A. *P < 0.05, **P < 0.01, ***P < 0.001, ****P < 0.0001

To determine whether inhibiting NPC1 can be therapeutic against cancers, we used the NPCI specific inhibitor U18666A to treat ARP-1, HepG2, and SKOV3 cells. The treatment led to increased levels of cleaved caspase-1 (Figures S6A–S6C) and noticeable cell swelling (Figure S6D), and an enhanced sensitivity of ARP-1 and HepG2 cells to nigericin (Fig. 4A). As expected, although NPC1 deficiency or U18666A treatment sensitized ARP-1 cells to nigericin-induced cell death, U18666A did not further enhance the sensitivity of NPC1-deficient cells to nigericin, which is consistent with the notion that NPC1 is the functional target of U18666A (Figure S6E). In addition, deletion of caspase-1 reversed the cytotoxicity of U18666A (Figure S6F). Moreover, U18666A treatment resulted in significant cancer cell death in ARP-1, HepG2, and SKOV3 cells detected by PI staining (Fig. 6A, also see Fig. 4A and S7A-S7B), while pyroptosis inhibitors effectively mitigated U18666A-induced cell death in ARP-1 and HepG2 cells (Figure S7C). U18666A also significantly inhibited the expression level of Ki67 in ARP-1, HepG2, and SKOV3 cells (Figures S7D-S7F), and suppressed cell growth in multiple cancer types (Figure S7G). Finally, U18666A impaired the clonogenic capacity of ARP-1, HepG2, and SKOV3 cells (Figs. 6B, S7H-S7I), supporting its anti-tumor efficacy in vitro.

To assess the therapeutic potential of U18666A in vivo, we employed human cancer cell xenograft mouse models. In an ARP-1 xenograft MM model, U18666A treatment significantly reduced tumor burdens (Fig. 6C–E). Similarly, in HepG2 and SKOV3 xenograft models, U18666A markedly reduced tumor burdens (Fig. 6F–J). We also explored the therapeutic efficacy of U18666A in the spontaneous VK*myc immune-competent MM mouse model [28]. After natural progression to MM, VK*myc mice were stratified based on disease severity and treated with PBS or U18666A. (Figure S8A). U18666A-treated mice exhibited reduced gamma globulin and total IgG levels (tumor burden) (Figs. 6K, S8B, and 6L) and had prolonged survival (Fig. 6M). Consistently, the number of cleaved caspase-1-positive MM cells from VK*myc mice was significantly increased after U18666A treatment (Figures S8C-D), and U18666A treatment also increased the production of HMGB1, IL-18, and IL-1β in the bone marrow (Fig. 6N). Importantly, U18666A was well-tolerated, without significant changes in body weight compared to PBS-treated mice (Figure S8E). Moreover, compared to ARP-1 multiple myeloma cells, peripheral blood mononuclear cells were less sensitive to U18666A (Figure S8F). These findings suggest that U18666A has a potential of a novel anticancer therapeutic agent.

**Fig. 7:**
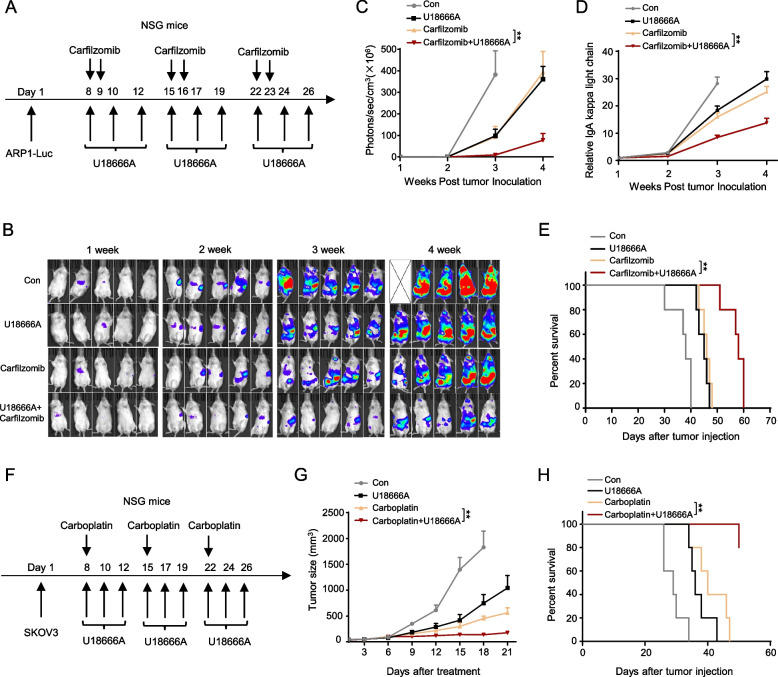
Effects of combination therapy using chemotherapeutics and U18666A against cancer. Flowchart of treating male NSG mice bearing ARP-1-luc cells with 5 mg/kg U18666A combined with 1 mg/kg carfilzomib (**A**). Bioluminescent images (**B**) and summarized results of bioluminescent signals of ARP-1-luc (**C**) cell-bearing NSG mice. ELISA was used to detect the levels of IgA kappa light chain in ARP-1 cell-bearing mice (**D**). Survival of ARP-1-luc cell-bearing NSG mice (**E**). Flowchart of treating female NSG mice bearing SKOV3 cells treated with 5 mg/kg U18666A combined with 30 mg/kg carboplatin (**F**); tumor sizes (**G**) and survival throughout the process (**H**). **P < 0.01 and ***P < 0.001

Finally, we wanted to determine whether NPC1 inhibitors can be used to treat cancers in combination with first-line chemotherapeutics. We first tested the efficacy of combining U18666A with the proteasome inhibitor carfilzomib in ARP-1 cells, the anthracycline drug epirubicin (EPI) in HepG2 cells, or the platinum-based drug carboplatin in SKOV3 cells. As expected, U18666A exhibited a strong additive effect with each of these chemotherapy agents in killing cancer cells in vitro (Figure S9A-S9C). Next, we tested these combinations in vivo. In MM mouse model, U18666A combined with carfilzomib significantly reduced tumor growth evident by the lowest luciferase signal intensity and IgA kappa chain levels in the combination treatment group (Fig. 7A–D) and extended mouse survival (Fig. 7E). Similarly, in the ovarian cancer model, combination of U18666A and carboplatin resulted in the smallest tumor burden (Fig. 7F-G) and significantly prolonged survival (Fig. 7H). Importantly, in both models, the combination treatments were well-tolerated, without significant impact on body weight of tumor-bearing mice (Figures S9D–S9E). These findings suggest that NPC1 inhibitors such as U18666A combined with conventional chemotherapy agents may offer enhanced therapeutic benefits for human cancers (Figure S10).

## Discussion

Cholesterol metabolism in tumors is reprogrammed, with tumor cells exhibiting a heightened dependence on cholesterol [50]. Cancer cell addiction to cholesterol requires high level of LDL uptake or enhanced cholesterol de novo synthesis. While several studies [35, 51], including ours [31], have highlighted the importance of the MVA pathway in tumor survival, the exact role of cholesterol in tumor cell survival has not yet been fully clarified. On one hand, tumor cells uptake large amounts of LDL from the environment and convert it into cholesterol [7]; on the other hand, cancer cells synthesize cholesterol through the MVA pathway to support their growth [52]. Despite these well-documented phenomena, the underlying mechanisms for cholesterol reprogramming in cancer remain unclear. In addition to cholesterol, the end product of the MVA pathway also includes GGPP, which has been shown to play a crucial role in conferring resistance to pyroptosis in tumor cells [35]. Under pyroptosis stress, tumor cells rely on GGPP synthesis to protect themselves from cell death, which may partly explain the reprogramming of cholesterol metabolism in these cells. Although previous studies have linked NPC1 to cholesterol-regulated cell fate [53, 54], these studies mainly focused on normal cells. Our work uncovers a novel mechanism by which NPC1 controls cell fate through cholesterol metabolism in tumor cells.

Previous studies showed that LDL uptake is tightly regulated by LDLR, which is ubiquitously expressed and mediates the internalization of plasma LDL-cholesterol [55–57]. However, in contrast to NPC1, LDLR is not commonly upregulated in tumors. In this study, we found that tumor cells preferentially upregulated NPC1 rather than LDLR to enhance LDL uptake under pyroptotic stress. While LDL-cholesterol uptake remains LDLR-dependent, the rate of uptake was significantly influenced by NPC1 expression in cancer cells. Analysis of cancer patient datasets revealed that higher NPC1 expression correlated with poorer patient survival, indicating that NPC1-regulated LDL-cholesterol uptake plays a critical role in tumor progression. These findings provide new insights into the complex relationship between pyroptosis, NPC1 function, and cholesterol metabolism in cancer cells.

Pyroptosis is one of the extensively studied forms of cell death in recent years, yet our understanding of its underlying mechanisms remains limited. Most studies have focused on how pyroptosis occurs, such as the cleavage activation of caspase-1 and the cleavage of GSDMD, which leads to pore formation in the cell membrane and ultimately cell death [16, 17, 19]. While it is known that cancer cells are more susceptible to pyroptosis, the mechanisms by which they survive under pyroptotic stress remain poorly understood. We discovered that, in response to pyroptosis pressure, cancer cells actively absorbed LDL to increase cellular cholesterol level, which in turn facilitated the synthesis of GGPP. This finding not only explains why cancer cells are "addicted" to cholesterol but also sheds light on the mechanism by which cancer cells resist pyroptosis.

It is well-established that p53 can influence various forms of programmed cell death, including pyroptosis [58–62]. In our study, we used a panel of cancer cell lines with diverse p53 backgrounds, including p53-null (SKOV3, ARP-1), wild-type p53 (HepG2, HCT116), and mutant p53 (RPMI-8226, MDA-MB-231). Despite these differences, NPC1 depletion consistently led to increased pyroptotic cell death. Thus, these findings suggest that NPC1’s role in modulating pyroptosis is likely to be at least partially independent of p53 status. Some studies showed induction of cell pyroptosis by combining nigericin with other agents such as ATP [63]. However, increasing evidence shows that nigericin alone can trigger pyroptosis—especially via the NLRP3–caspase‑1–GSDMD pathway [35, 64]. Consistently, our results demonstrated that nigericin treatment combined with NPC1 depletion significantly exacerbated pyroptosis, highlighting the critical role of NPC1 in this pathway.

Chemotherapy is the most widely used and effective treatment for human cancers; however, its efficacy is limited by the fact that each chemotherapeutic drug targets only specific types of cancer, and some patients do not respond to treatment, ultimately leading to poor outcomes [65]. Therefore, identifying new therapeutic targets for cancers and developing new drugs targeting these pathways are critical to improving patient outcomes. Although studies have demonstrated that targeting pyroptosis is an effective approach for cancer treatment [24, 26, 66], there are no FDA-approved therapies aimed at modulating pyroptosis in cancer. Our research demonstrates that the NPC1 inhibitor U18666A can effectively target NPC1 to induce pyroptosis in cancer cells. This study highlights the potential of U18666A and other NCP1 inhibitors as novel chemotherapeutic agents for cancer treatment by inducing tumor pyroptosis. Moreover, combining U18666A with traditional chemotherapy agents showed enhanced efficacy. These findings suggest that U18666A holds a significant clinical translational potential as treatments for cancer by targeting NPC1 to induce pyroptosis in cancer cells.

## Conclusions

In this study, we analyzed cancer patient datasets and observed that high expression of NPC1 was closely associated with an aggressive behavior of tumors and poor survival rates, indicating that NPC1 may play an important role in tumor pathogenesis and serve as a promising therapeutic target. Consistent with these findings, NPC1 deficiency significantly inhibited tumor progression by enhancing pyroptosis in cancer cells. NPC1 was essential for the uptake of LDL by tumor cells. Within cells, LDL is degraded to release free cholesterol, which serves as a critical resource for cellular processes. The terminal products of the MVA pathway, cholesterol and GGPP, are both indispensable for cancer cells. Cholesterol supports the rapid proliferation of cancer cells [5], while GGPP enables them to resist pyroptosis [35].

We demonstrated that under pyroptosis stress conditions, tumor cells became increasingly dependent on cholesterol absorption and shifted their demand to GGPP synthesis instead of cholesterol to resist pyroptosis. This reprogramming of cholesterol metabolism through the upregulation of NPC1 expression was critical for the survival of cancer cells. We observed that targeting NPC1 to reverse this metabolic reprogramming represents an effective strategy for killing cancer cells. To our knowledge, we are the first to demonstrate that targeting NPC1 in the cholesterol absorption pathway of cancer cells not only can induce pyroptosis but also reduce tumor burden in vivo. Furthermore, our data suggest that U18666A, when combined with conventional chemotherapy agents such as carfilzomib in MM or carboplatin in ovarian cancer, may provide enhanced therapeutic benefits. Thus, our research reveals that NPC1 protects cancer cells from pyroptosis by reprogramming tumor cholesterol metabolism and identifies NPC1 as a therapeutic target for human cancer.

## Supplementary Information

Supplementary Material 1.

Supplementary Material 2.

## Data Availability

No datasets were generated or analysed during the current study.

## Abbreviations

LDL: Low-density lipoprotein
NPC1: Niemann-Pick C1
LDLR: LDL receptor
MM: Multiple myeloma
HCC: Hepatocellular carcinoma
KRCC: Kidney renal cell carcinoma
LFM: Lipid-free medium
MVA: Mevalonate
GGPP: Geranylgeranyl pyrophosphate
