# Supplementary figures and images for: NPC1 as a novel therapeutic target for induction of pyroptosis in cancers
Source: Biomark Res. 2025 Sep 26;13:115. doi: 10.1186/s40364-025-00823-w (PMC12465410; doi:10.1186/s40364-025-00823-w)

Figure S1

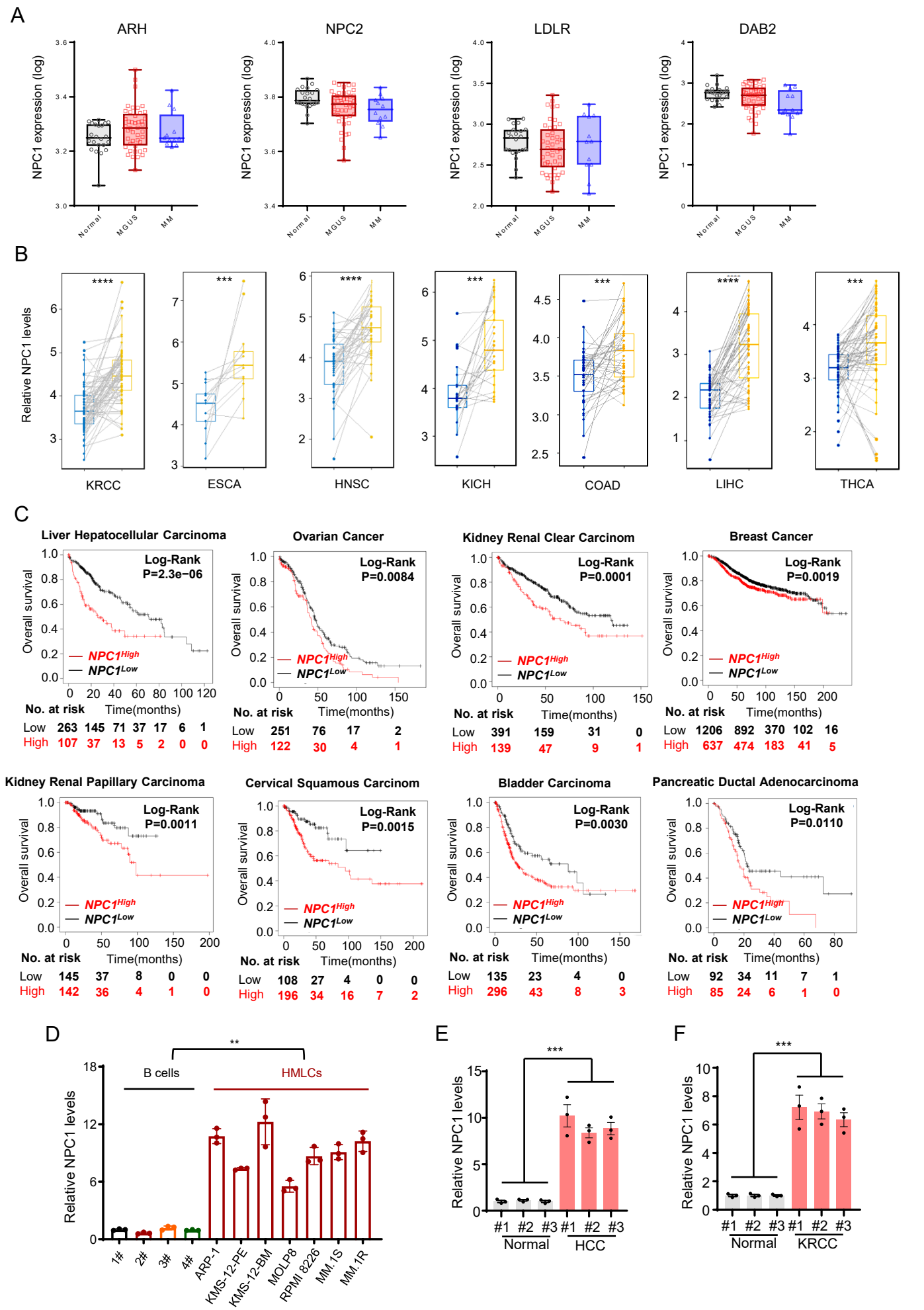

Figure S2

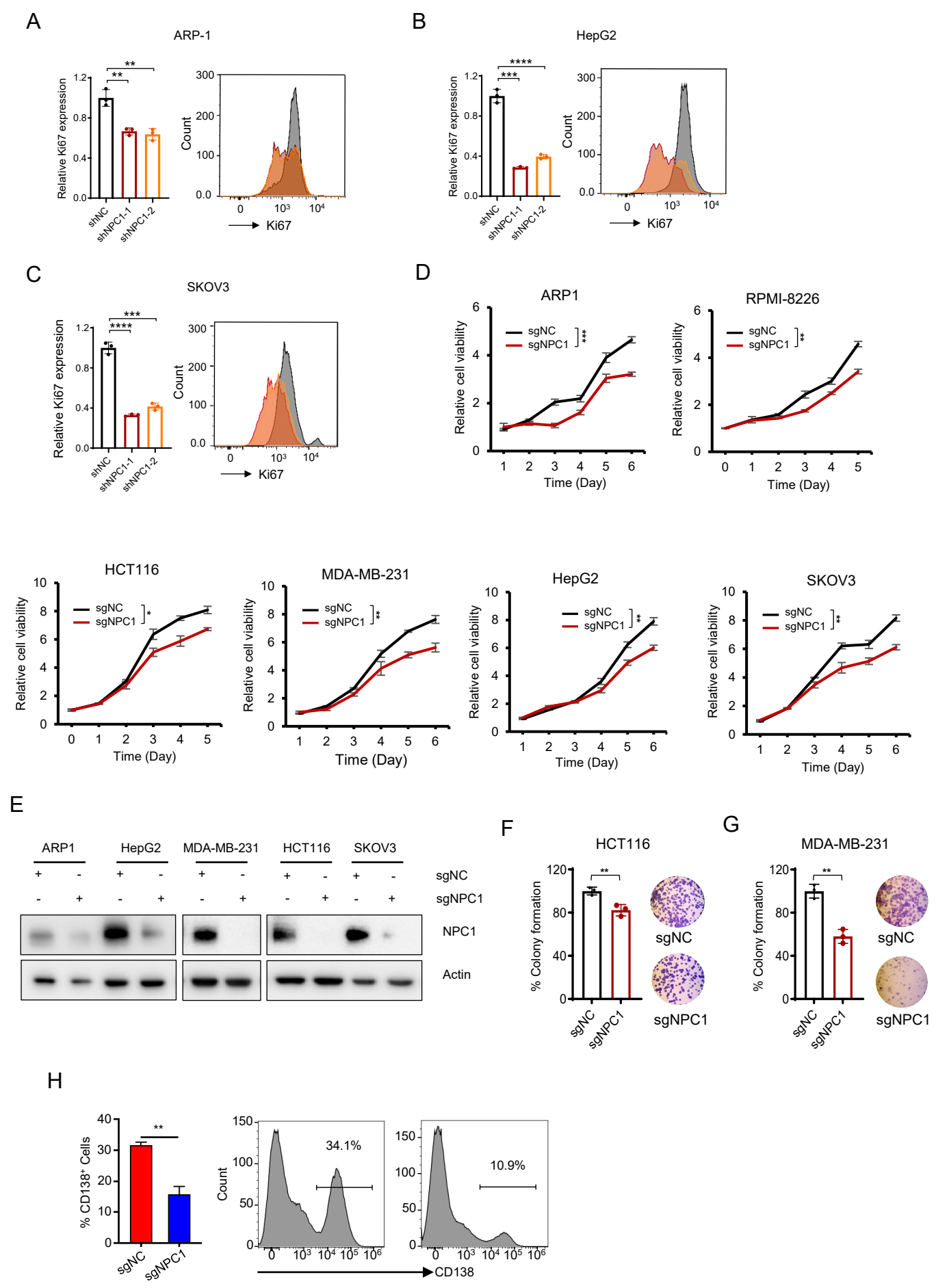

Figure S3

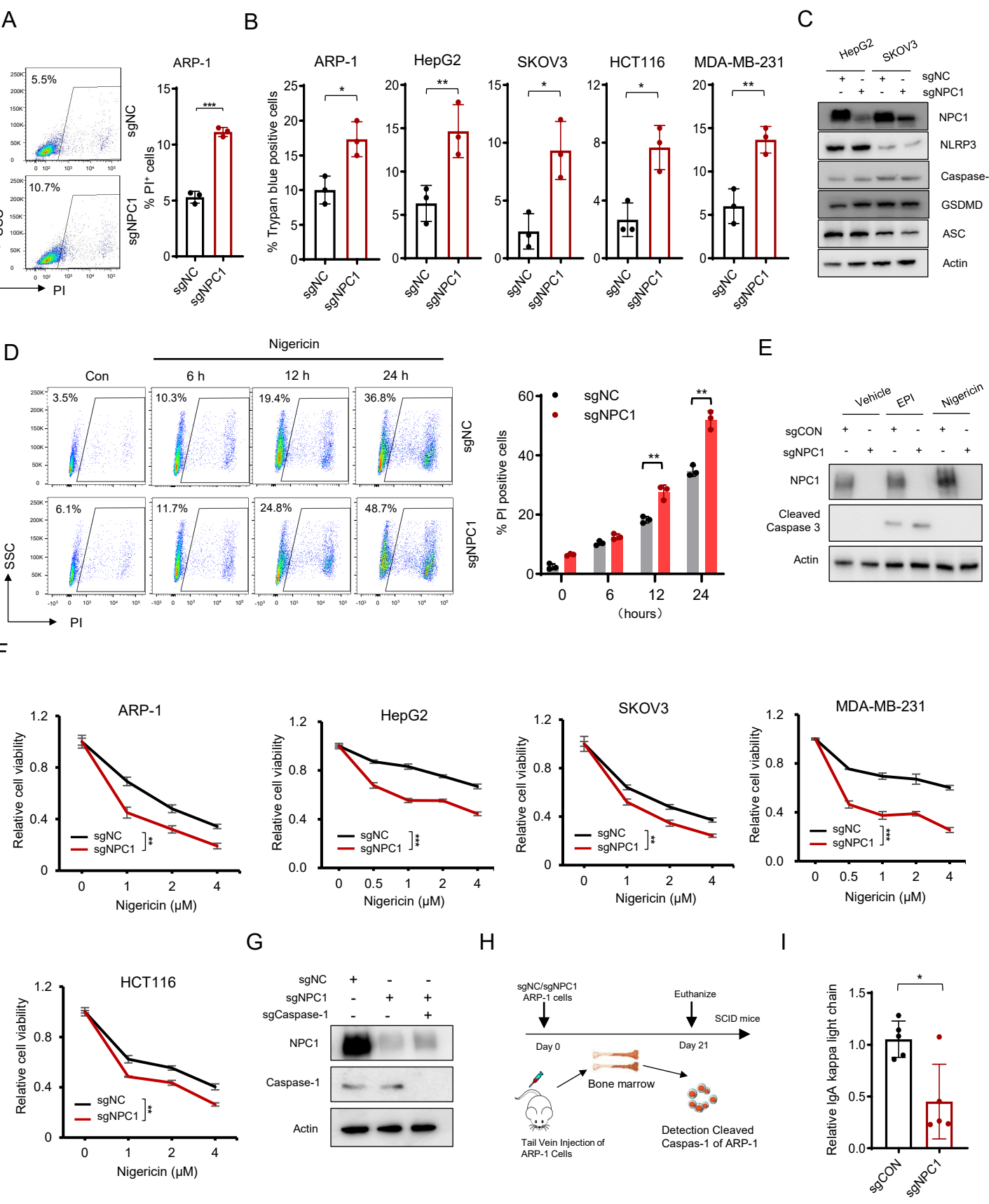

Figure S4

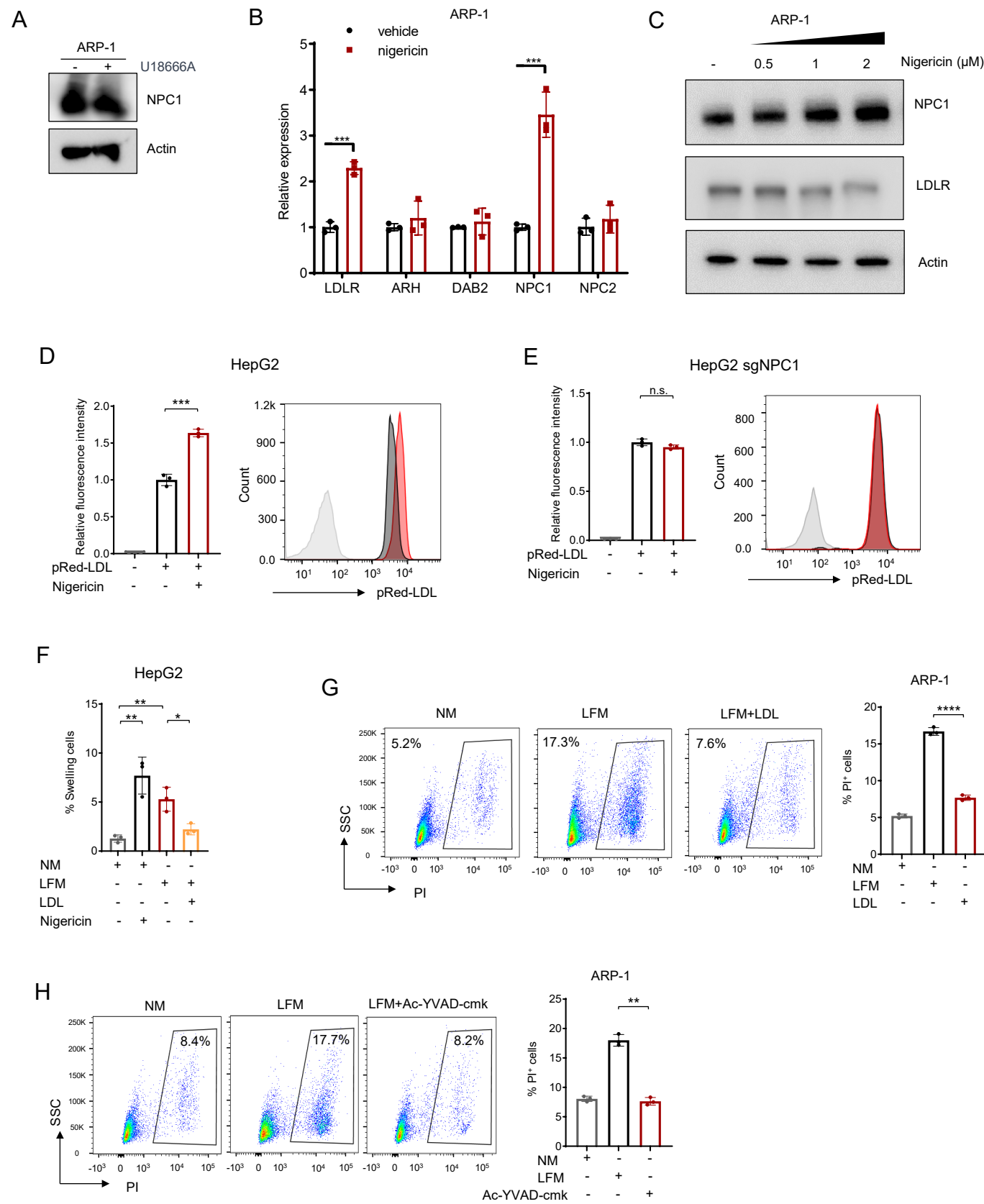

Figure S5

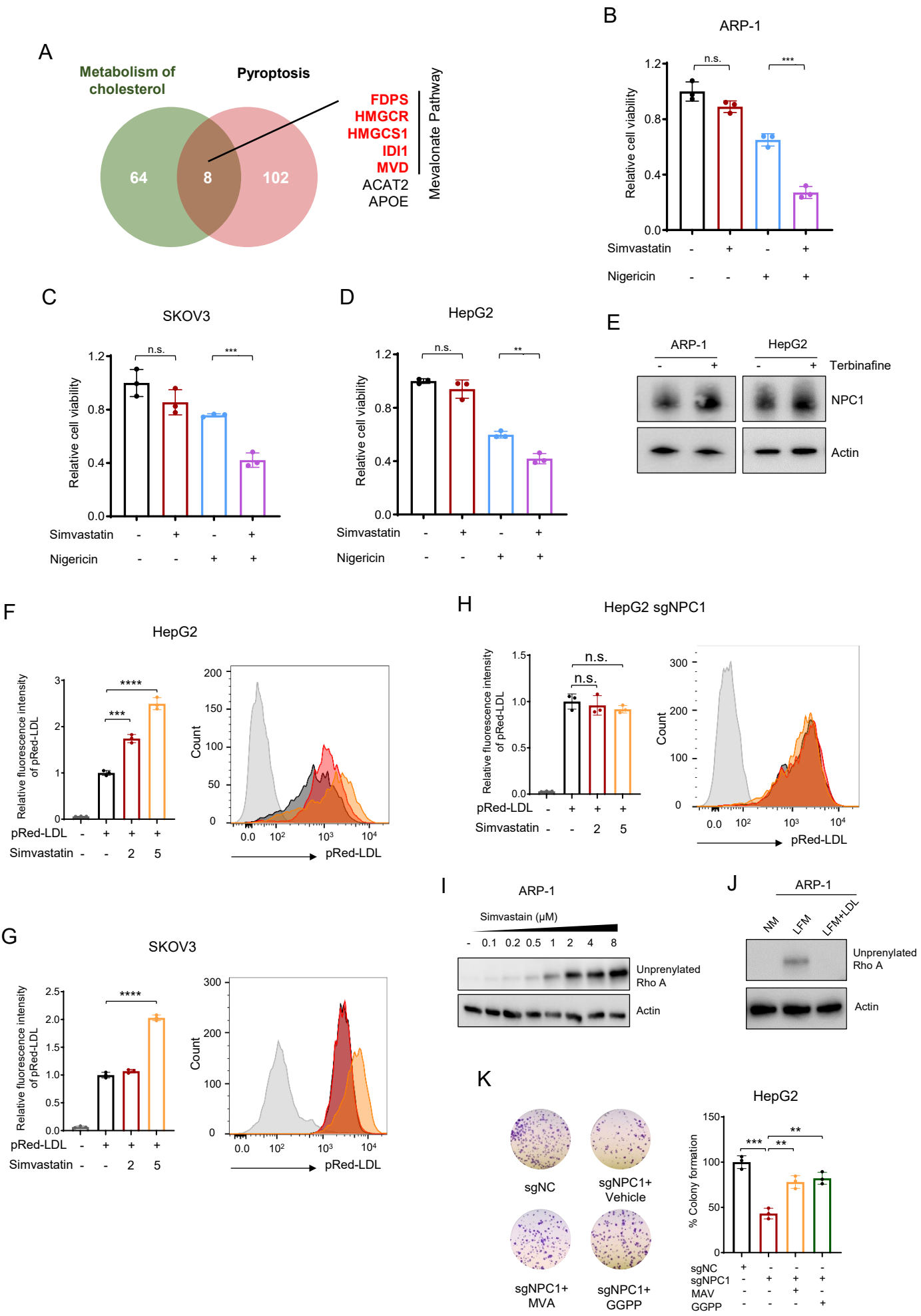

Figure S6

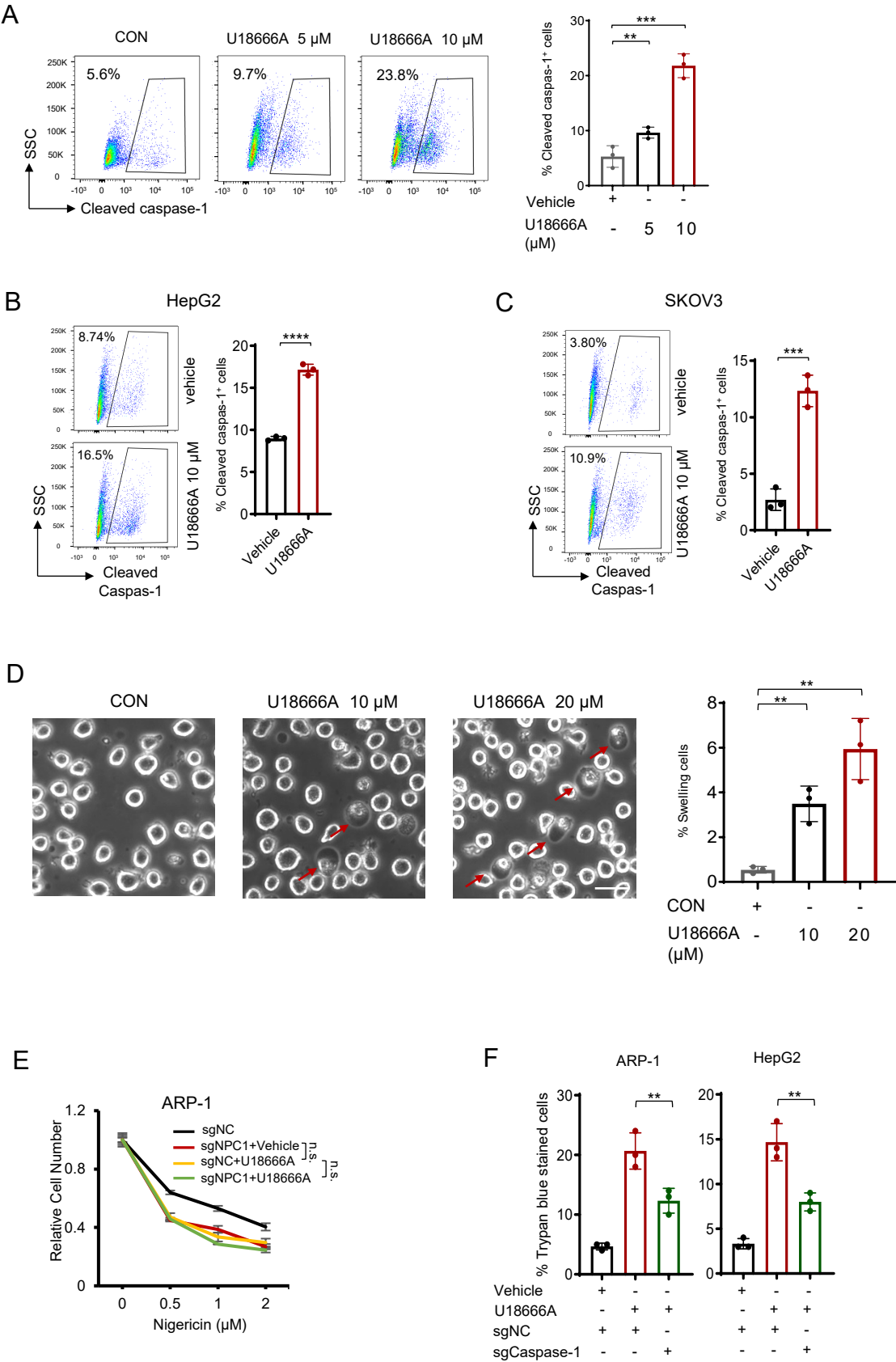

Figure S7

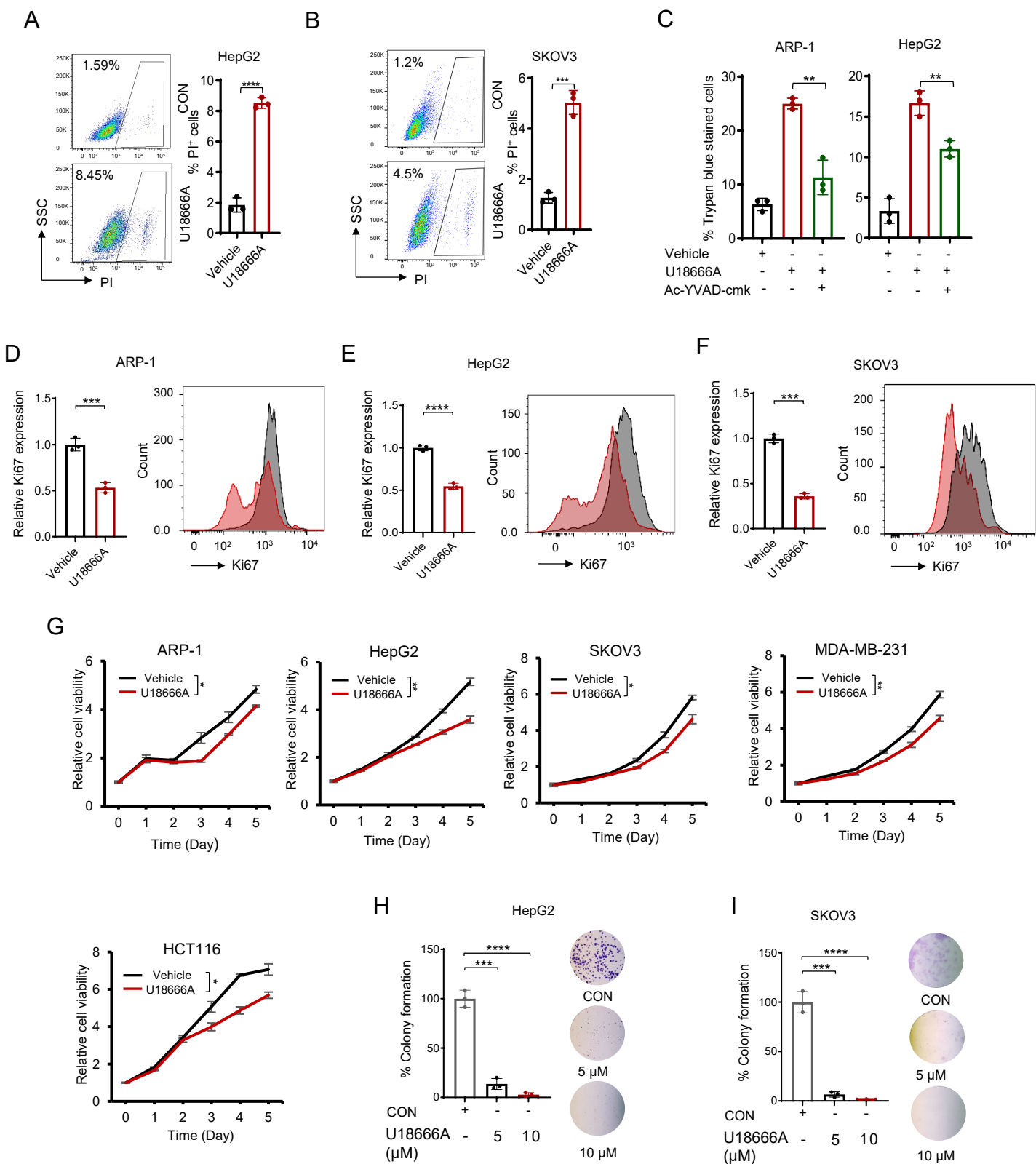

Figure S8

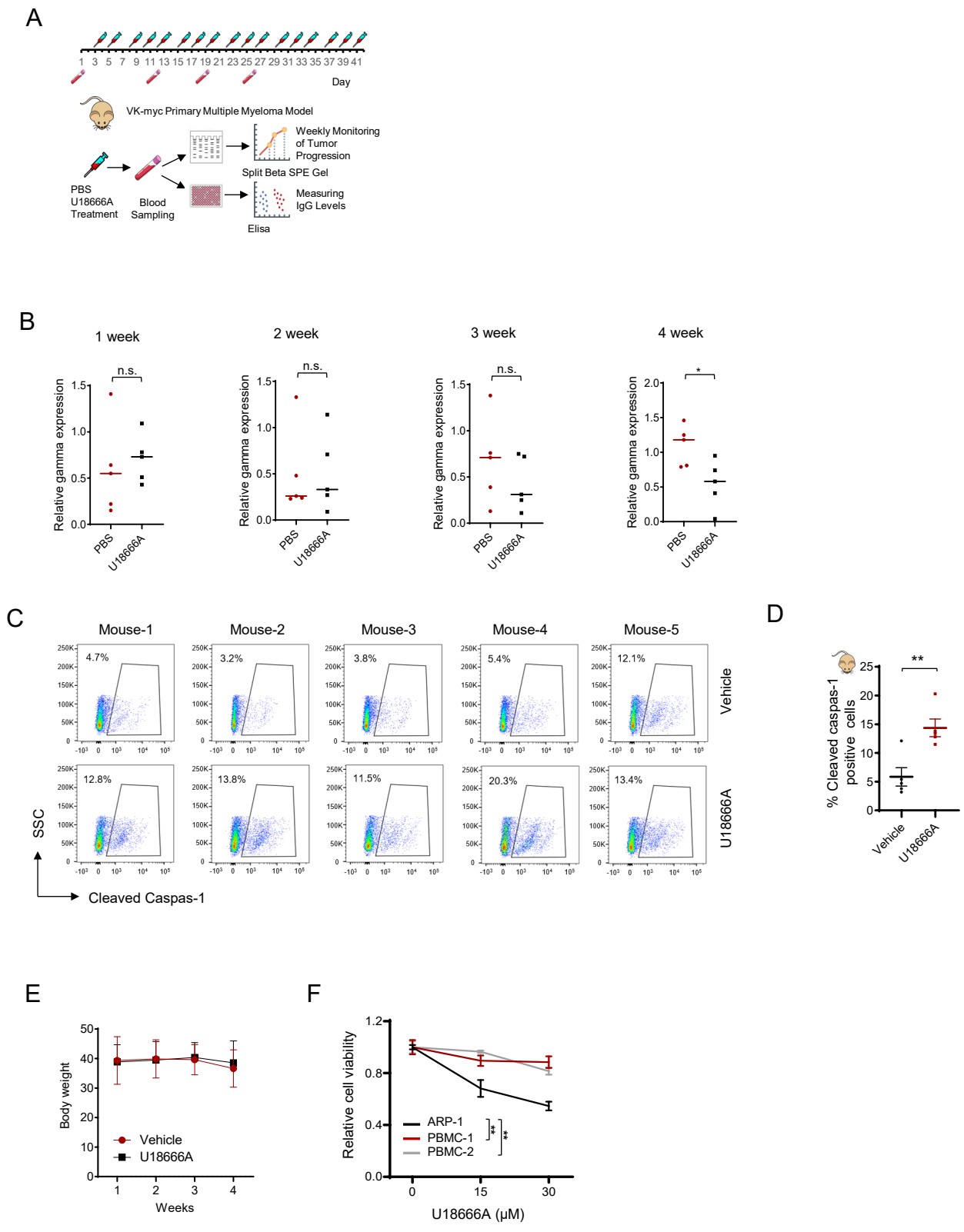

Figure S9

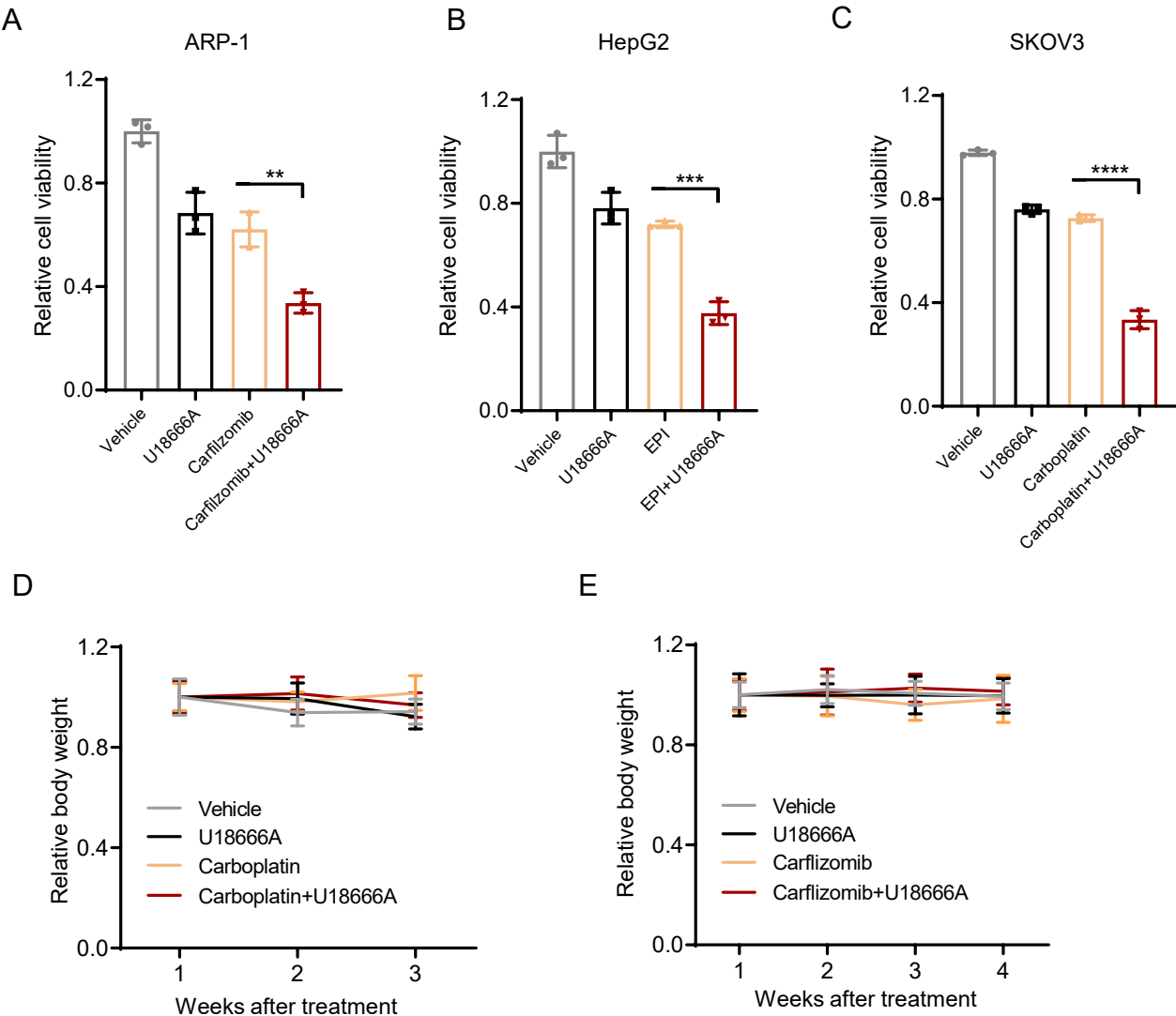

Figure S10

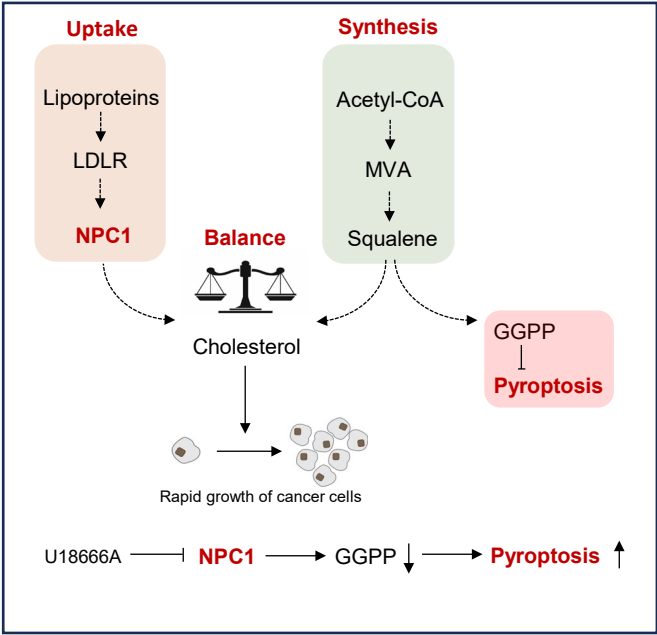

Supplement: Supplementary file 1 — Supplementary Material 1. [file 40364_2025_823_MOESM1_ESM.pdf]
