# Supplementary material for: NPC1 as a novel therapeutic target for induction of pyroptosis in cancers
Source: Biomark Res. 2025 Sep 26;13:115. doi: 10.1186/s40364-025-00823-w (PMC12465410; doi:10.1186/s40364-025-00823-w)
Supplement: Supplementary file 2 — Supplementary Material 2. [file 40364_2025_823_MOESM2_ESM.docx]

**Supplementary Figure 1. NPC1 is upregulated in various cancers and correlates with poor prognosis.**
Expression of *ARH, NPC2, LDLR, DAB2* mRNA in plasma cells of normal individual (n = 22) and patients with MGUS (n = 44) or MM (n = 12) in Zhan MM dataset **(A)**. TCGA database showing *NPC1* mRNA expression in different cancers and their corresponding normal tissues KRCC: kidney renal clear cell carcinoma; ESCA: esophageal carcinoma; HNSC: head and neck squamous cell carcinoma; KICH: kidney chromophobe; COAD: colon adenocarcinoma; LIHC: liver hepatocellular carcinoma; THCA: thyroid carcinoma. **(B)**. The KM-plot database showing the relationship between NPC1 expression and overall survival in various cancer types **(C)**, the "Auto select best cutoff percentile" function was used. qPCR analysis of *NPC1* mRNA expression in normal human B cells and MM cell lines **(D)**. qPCR analysis of NPC1 mRNA expression in HCC and KRCC, as well as their respective normal tissues **(E)**. **P < 0.01, ***P < 0.001, ****P < 0.0001.

**Supplementary Figure 2.** **NPC1 deficiency slows cancer growth in vitro and in vivo.**
After transfection with the indicated sgRNAs, the growth of ARP-1, HepG2, RPMI-8226, SKOV3, HCT116 and MDA-MB-231 cells was measured by cell viability assay **(A)**. Western blot showing the knockdown efficiency of sgRNA **(B)**. Flow cytometry (summarized results in left panel and flow staining in right) analysis of Ki67 expression in ARP-1 **(C)**, HepG2 **(D)**, and SKOV3 **(E)** cells after knocking down NPC1. Results (representative images and summarized results) of colony formation in HCT116 **(F)**, and MDA-MB-231 **(G)** cells after transfection with control (CON) or NPC1 sgRNA. Bone marrow cells were extracted from NSG mice bearing ARP-1 and ARP-1-NPC1-depleted cells and flow cytometry (summarized results in left panel and flow staining in right) analysis of CD138-positive cells **(H)**. *P < 0.05, **P < 0.01, ***P < 0.001, ****P < 0.0001.

**Supplementary Figure 3. NPC1 protects cancer cells from pyroptosis.**
Percentages (representative flow images [left panels] and summarized results [right panel]) of PI^+^ ARP-1cells expressing NPC1 sgRNA examined by flow cytometry **(A)**. Trypan blue staining showing the proportion of dead cells in ARP-1, HepG2, SKOV3, HCT116, and MDA-MB-231 cells expressing control (CON) or NPC1 sgRNA **(B)**. Western blot showing the levels of NLRP3, caspase-1, GSDMD and ASC in NPC1-depleted HepG2 and SKOV3 cells **(C)**. Percentages (representative flow images [left panels] and summarized results [right panel]) of PI^+^ ARP-1 cells following the treatment with 2 μM nigericin at the indicated time points examined by flow cytometry **(D)**. Western blot showing the levels of cleaved caspase-3 in NPC1-depleted ARP-1 cells treated with epirubicin (EPI) and nigericin. EPI serves as a positive control for inducing cleaved caspase-3 (apoptosis) **(E)**. Cell viability assay showing the survival of ARP-1 cells transfected with the control (CON) or NPC1 sgRNA and treated with the indicated doses of nigericin **(F)**. Western blot showing the levels of NPC1 and caspase-1 in cells expressing the indicated sgRNAs **(G)**. Experimental design for in vivo analysis of cleaved caspase-1 activity in NPC1-depleted ARP-1 cells in mice **(H)**. ELISA showing the levels of IgA kappa light chain (as a measure of tumor burden) in mice bearing ARP-1 cells expressing sgNC or sgNPC1 **(I)**. *P < 0.05, **P < 0.01, ***P < 0.001.

**Supplementary Figure 4. Cancer cells require NPC1 for an enhanced LDL uptake to resist pyroptosis.**Western blot showing NPC1 expression in ARP-1 cells before and after treatment with U18666A (5 µM, 24 hours) **(A)**. qPCR analysis of the expression of genes involved in the LDL uptake pathway in ARP-1 cells after treatment with Nigericin (1 µM, 24 hours) **(B)**. ARP-1 cells were treated with indicated doses of Nigericin for 24 hours, and western blot was used to detect the changes in NPC1 and LDLR expression **(C)**. Fluorescence intensity (summarized results in left panel and flow staining in right) of HepG2 **(D)** and NPC1-depleted HepG2 **(E)** cells treated with 1 μM nigericin in the presence of fluorescence-labeled pRed-LDL for 24 hours. Summarized results of HepG2 cells showing cell swelling after continuous culture in NM, LFM, and LFM+LDL (20 μg/ml) for three days. Cells treated with 1 μM nigericin for 24 hours served as a positive control. The proportion of cell swelling was observed in > 200 cells **(F)**. ARP-1 cells cultured in NM, LFM, and LFM+LDL (20 μg/ml) medium examined by flow cytometry and the percentages of PI-stained ARP-1 cells are shown **(G)**. ARP-1 cells cultured in NM, LFM, and LFM+ Ac-YVAD-cmk (20 μM) medium examined by flow cytometry and the percentages of PI-stained ARP-1 cells are shown **(H)**. *P < 0.05, **P < 0.01, ***P < 0.001, ****P < 0.0001.

**Supplementary Figure 5. Pyroptosis in NPC1-depleted cells is mediated by the mevalonate pathway metabolite.**
The intersection of genes between the cholesterol metabolism pathway and the pyroptosis pathway **(A)**. Cell viability assay on ARP-1 **(B)**, HepG2 **(C)** and SKOV3 **(D)** cells, with or without simvastatin (5 µM) or nigericin (1 µM) for 24 hours. Western blot showing NPC1 expression in ARP-1 and HepG2 cells before and after treatment with Terbinafine (20 μM) for 24 hours **(E)**. Fluorescence intensity (summarized results in left panel and flow staining in right) of HepG2 **(F)**, SKOV3 **(G)** and NPC1-depleted HepG2 **(H)** cells treated with indicated concentration of simvastatin in the presence of fluorescence-labeled pRed-LDL for 24 hours. Western blot showing the changes in unprenylated Rho A in ARP-1 cells after treatment with specific concentrations of simvastatin **(I)**. Western blot showing the changes in unprenylated Rho A in ARP-1 cells after 3 days of treatment with NM, LFM, and LFM + LDL (20 μg/ml) **(J)**. Clonogenic activity of NPC1-depleted HepG2 cells, with or without MAV (100 µM) or GGPP (10 µM) **(K)**. **P < 0.01, ***P < 0.001, ****P < 0.0001.

**Supplementary Figure 6. U18666A induces pyroptosis in cancer cells.**
ARP-1 cells were treated with indicated concentrations of U18666A for 48 hours and examined by flow cytometry. The percentages of cleaved caspas-1-postived ARP-1 cells are shown **(A)**. HepG2 **(B)** and SKOV3 **(C)** cells were treated with 10 µM U18666A for 48 hours and examined by flow cytometry. The percentages of cleaved caspas-1-postived ARP-1 cells are shown. Representative images (left panels) and summarized results (right panel) of ARP-1 cells showing cell swelling after continuous culture in indicated concentrations of U18666A for 48 hours with > 200 cells counted for each group, scale bar = 30 µm **(D)**. Cells were treated with indicated concentrations of nigericin, with or without U18666A (5 µM). Cell viability showing the survival rate **(E)**. Trypan blue staining was used to assess the proportion of dead cells before and after U18666A (5 µM) treatment in cells with NPC1 depletion or simultaneous deletion of NPC1 and caspase-1. **(F)**. **P < 0.01, ***P < 0.001, ****P < 0.0001.

**Supplementary Figure 7. Targeting NPC1 by U18666A is therapeutic against cancer.**
Percentages (representative images in left panels and summarized results in right) of PI-positive HepG2 **(A)** and SKOV3 **(B)** cells cultured with 2 µM U18666A for 72 hours and examined by flow cytometry. ARP-1 and HepG2 cells were treated with vehicle, U18666A (10 µM), or U18666A and Ac-YVAD-cmk (20 µM). Trypan blue staining showing the proportion of dead cells **(C)**. Flow cytometry showing Ki67 expression in ARP-1 **(D)**, HepG2 **(E)** and SKOV3 **(F)** cells treated with or without U18666A (10 µM) for 24 hours. Cell viability assay showing the growth of ARP-1, HepG2, SKOV3, MDA-MB-231 and HCT116 cells, with or without the addition of U18666A (5 µM) **(G)**. Clonogenic activity of HepG2 **(H)** and SKOV3 **(I)** cells, with or without the indicated dose of U18666A. *P < 0.05, **P < 0.01, ***P < 0.001, ****P < 0.0001.

**Supplementary Figure 8. Targeting NPC1 by U18666A in Vk*myc mice.**Flowchart of treating Vk*myc mice with U18666A (5 mg/kg) **(A)**. Tumor burdens detected by quantification of the SPE QuickGel Gamma region **(B).** Flow cytometry (representative flow images **[C]** and summarized results **[D]**) of cleaved caspase-1 levels in vehicle and U18666A treated MM cells in Vk*myc mice. Body weight of Vk*myc mice with or without U18666A treatment **(E)**. Cell viability assays were performed to evaluate the cytotoxic effects of U18666A on ARP-1 and peripheral blood mononuclear cells (PBMCs) **(F)**. *P < 0.05, **P < 0.01.

**Supplementary Figure 9. Chemotherapy combined with U18666A enhances the therapeutic efficacy against cancer.**
Cell viability assay showing the survival rate of ARP-1 cells after treatment with U18666A (10 µM), carfilzomib (4 nM), or their combination for 24 hours **(A)**. Cell viability assay showing the survival rate of HepG2 cells after treatment with U18666A (10 µM), EPI (1 µM), or their combination for 24 hours **(B)**. Cell viability assay showing the survival rate of SKOV3 cells after treatment with U18666A (10 µM), carboplatin (10 µM), or their combination for 24 hours **(C)**. Body weight of NSG mice bearing ARP-1 cells **(D)** and SKOV3 cells **(E)**. **P < 0.01, ***P < 0.001, ****P < 0.0001.

**Supplementary Figure 10. Schematic model of NPC1’s function in pyroptosis and tumor treatment via targeting NPC1 with U18666A.**Genetic and pharmacological inhibition of NPC1 disrupts cholesterol uptake, induces pyroptosis, and suppresses tumor progression in vitro and in vivo. U18666A, an NPC1 inhibitor, enhances chemotherapy efficacy in MM and solid tumors, highlighting its potential as a therapeutic agent.
